# Mesenchymal stem cell-derived extracellular vesicles protect against abdominal aortic aneurysm formation by inhibiting NET-induced ferroptosis

**DOI:** 10.1038/s12276-023-00986-2

**Published:** 2023-05-01

**Authors:** Liang Chen, Yuting Liu, Zheyu Wang, Leiyang Zhang, Yi Xu, Yinan Li, Lan Zhang, Guiming Wang, Shuofei Yang, Guanhua Xue

**Affiliations:** 1 Department of Vascular Surgery, Renji Hospital, School of Medicine, Shanghai Jiao Tong University, Pujian Road 160, 200127 Shanghai, China; 2 Department of Thoracic and Cardiovascular Surgery, Nanjing First Hospital, Nanjing Medical University, 210000 Nanjing, China; 3 Department of Vascular Surgery, The First Hospital of Shanxi Medical University, 030001 Taiyuan, China

**Keywords:** Aneurysm, Aortic diseases

## Abstract

Neutrophil extracellular traps (NETs) play an important role in abdominal aortic aneurysm (AAA) formation; however, the underlying molecular mechanisms remain unclear. Mesenchymal stem cell-derived extracellular vesicles (MSC-EVs) may exert therapeutic effects on AAA through their immunomodulatory and regenerative abilities. This study aimed to examine the role and mechanism of MSC-EVs in regulating the development of NET-mediated AAA. Excessive release of NETs was observed in patients with AAA, and the levels of NET components were associated with the clinical outcomes of the patients. Datasets from the Gene Expression Omnibus database were analyzed and revealed that the PI3K/AKT pathway and ferroptosis were strongly associated with NETosis during AAA formation. Further experiments verified that NETs promoted AAA formation by inducing ferroptosis in smooth muscle cells (SMCs) by inhibiting the PI3K/AKT pathway. The PI3K agonist 740 Y-P, the ferroptosis inhibitor ferrostatin-1, and *Padi4* deficiency significantly prevented AAA formation. MSC-EVs attenuated AAA formation by reducing NET release in an angiotensin II-induced AAA mouse model. In vitro experiments revealed that MSC-EVs reduced the release of NETs by shifting NETosis to apoptosis. Our study indicates an important role for NET-induced SMC ferroptosis in AAA formation and provides several potential targets for AAA treatment.

## Vascular disease: Stem cell packets help reduce artery bulge

Tiny membrane-bound packets of biomolecules secreted by stem cells in the bone marrow umbilical cord can reduce the development of dangerous bulges or ballooning in the aorta, the body’s largest artery. Liang Chen of China’s Shanghai Jiao Tong University School of Medicine and colleagues report that these stem cell-derived vesicles help to prevent such bulges, known as aneurysms, in mice through their effects on a type of immune cell called neutrophils, which release networks of DNA and protein at sites of inflammation. The neutrophil-produced molecules induced a type of cell death in the smooth muscle cells of the arterial wall, but the stem cell-secreted vesicles helped to suppress this process. These findings could lead to new therapies for people suffering from aortic aneurysm, a condition that can lead to life-threatening ruptures if left untreated.

## Introduction

Abdominal aortic aneurysm (AAA) is a fatal vascular disease that manifests as a local enlargement of the abdominal aorta^1^. At present, effective pharmacological treatments for AAA are urgently required in the clinic^2^. A better understanding of the cellular and molecular mechanisms underlying AAA formation is necessary for developing efficient pharmacotherapies for patients with AAA. Neutrophil extracellular traps (NETs) represent a host defense mechanism for catching and killing pathogens using a sticky extracellular network loaded with bactericidal proteins^3^. NETs are significantly associated with the formation of AAA^4^. The release of NETs begins with the activation of peptidyl arginine deiminase 4 (PAD4, encoded by *Padi4*), which causes histone citrullination, extensive chromatin decondensation, and nuclear localization of granular enzymes (e.g., myeloperoxidase and neutrophil elastase)^5^. Gut microbiome dysbiosis and IL-1β can lead to AAA development by inducing NETosis^6,7^. Inhibiting NET formation with PAD4 inhibitors or the decomposition of NETs by deoxyribonuclease I (DNase I) can significantly reduce AAA formation in experimental models^8,9^. However, the specific role of NETs in AAA remains unclear.

The depletion of smooth muscle cells (SMCs), which is a major pathological characteristic of AAA, is closely related to the initiation and development of AAA^10^. However, the critical inflammatory pathways that lead to the depletion of SMCs during AAA formation are unclear. Ferroptosis is a form of programmed cell death characterized by high iron-dependent lipid peroxidation^11^. Ferroptosis in SMCs is associated with aortic dissection, and ferroptosis inhibitors can protect against aortic dissection^12^. Ferroptosis and related genes play critical roles in the formation of AAA and may serve as promising targets in the treatment of AAA^13^. NETs can induce ferroptosis in alveolar epithelial cells during acute lung injury^14^. Moreover, NET-induced apoptosis in SMCs plays a critical role in AAA rupture^9^. However, the intrinsic relationship between NETs and SMC ferroptosis during AAA formation remains unknown.

Due to their immunomodulatory and regenerative abilities, mesenchymal stem cells (MSCs) have shown favorable therapeutic efficacy in suppressing AAA formation by reducing the degradation of aortic elastin^15^. However, the specific mechanisms underlying the therapeutic effects of MSCs remain unknown. Mesenchymal stem cell-derived extracellular vesicles (MSC-EVs) have emerged as critical cellular components that facilitate the therapeutic effects of MSCs^16^. However, the effects of MSC-EVs on AAA remain unclear. A recent study revealed the antiproteolytic and matrix-regenerating effects of extracellular vesicles extracted from human bone marrow-derived MSCs in proteolytic injury-induced cultures of SMCs isolated from rats with AAA^17^. In addition, MSC-EVs can inhibit NET formation by restoring the mitochondrial function of neutrophils^18^. The dysfunction of SMCs and neutrophils is an important cause of AAA formation; therefore, the regulatory effects of MSC-EVs on SMCs and NETs warrant in-depth investigation. This study aimed to examine the specific mechanisms underlying the regulatory role of MSC-EVs in NET-mediated AAA formation.

## Materials and methods

### Human studies

Patients undergoing open aortic surgery were recruited according to the study protocol approved by the Institutional Review Board of the Shanghai Jiao Tong University School of Medicine, Renji Hospital (No. KY2021–216). All experiments involving human participants were conducted in accordance with the principles of the Declaration of Helsinki. All patients or their proxies signed a written informed consent form before the specimens were collected. Before surgery, the presence of an AAA was documented via echocardiography or computed tomography/magnetic resonance imaging, and a clinical diagnosis was established by expert clinicians. Healthy aorta samples were obtained from patients who were brain-dead or organ donors who did not have an aneurysm or other cardiovascular diseases. The baseline demographic information, medical history, risk factors, biochemical and hematological data, clinical outcomes, and follow-up information were collected and are presented in Supplementary Table 1.

### Animal models and the assessment of aneurysm formation

Apolipoprotein E-deficient (*ApoE*^−/−^) C57BL/6J mice used in this study were handled in accordance with the Guide for the Care and Use of Laboratory Animals guidelines published by the National Institutes of Health (NIH Publication No. 8023, revised 1978). The mice were purchased from the Shanghai Experimental Animal Center and were housed at the Animal Science Center, School of Medicine, Shanghai Jiao Tong University (12-h light/dark cycle at 22 °C, two mice per cage). All experiments involving mice were approved by the Ethical Review Committee (No. KY2021–216). At the end of the experiments, all animals were anesthetized via isoflurane inhalation (2–5%) and euthanized via exsanguination and bilateral thoracotomy.

*ApoE*^−/−^ mice were crossbred with C57BL/6J *Padi4*^−/−^ mice to produce *ApoE*^+/−^*Padi4*^+/−^ mice, which were then backcrossed with *ApoE*^−/−^ mice to produce *ApoE*^−/−^*Padi4*^+/−^ mice, which were intercrossed to produce *ApoE*^−/−^*Padi4*^−/−^ mice. To determine the genotypes of the mice, tail samples were collected for polymerase chain reaction (PCR).

#### Construction of mouse models of AAA

For angiotensin II (AngII) infusion, the mice were anesthetized via isoflurane inhalation (induction dose: 3–5%, maintenance dose: 1–2%). Mouse models of Ang II-induced AAA were generated as follows: micro-osmotic pumps (ALZET DURECT 1004, Durect Corp^−1^, Cupertino, CA, USA) containing saline (isotonic sodium chloride solution) or Ang II (A9525, Sigma‒Aldrich, St. Louis, MO, USA) were subcutaneously implanted into 12-week-old male mice and administered a dose of 1000 ng/(kg.min) for 28 days. To determine the therapeutic effects of MSC-EVs, MSC-EVs (100 μL of PBS containing ~7 × 10^10^ particles) were injected into the mice via the tail vein on Day 1. Control mice were injected with 100 μL of PBS. To modulate NETs, the PI3K/AKT pathway or ferroptosis in vivo, DNase I (10 mL/kg/d, InvivoGen, San Diego, CA, USA), 740 Y-P (10 mg/kg/d, MedChemExpress, USA), ferrostatin-1 (Fer-1, 0.8 mg/kg/d, MedChemExpress, USA) or a drug vehicle was administered intravenously every other day starting 1 day before Ang II infusion. Hematoxylin and eosin (H&E) staining of aortic tissue samples and ultrasonic imaging were performed to verify the successful establishment of the mouse models (Supplementary Fig. 1a, b).

The aortic diameter was examined via echocardiography. The mice were anesthetized via isoflurane inhalation (induction dose: 3–5%). After the mice were sedated, anesthesia was maintained with 1% isoflurane during the examination using an ultrasound device equipped with a 40-MHz transducer (Visual Sonics Vevo 2100, FUJIFILM, USA). B-mode ultrasonography was used to identify the abdominal aorta. Color and spectral Doppler ultrasonography were used to verify that the direction and velocity of blood flow were consistent with the aortic signal. Images of the abdominal aortas of mice were captured, and the maximal aortic diameter was measured as the internal diameter using statistical software. AAA was defined as ≥50% dilatation of the outer diameter of the abdominal aorta compared with the normal outer diameter of the abdominal aorta. The tissues were subjected to pathological assessments and other biochemical tests.

### Morphological and pathological assessments

The aorta was observed under a dissecting microscope, and images were captured after the removal of surrounding tissues (Leica DM6000B microscope; Wetzlar, Germany). The maximal aortic diameter and total aortic area were measured using inbuilt analysis software by Leica (LAS X software, Version 2017.3.6). Subsequently, the abdominal aorta was harvested, fixed in 4% paraformaldehyde (PFA), and embedded in paraffin. Tissue sections (5 μm) were stained with H&E or Verhoeff’s elastic (EVG) stain using a commercial kit (DAKO, Denmark) according to the manufacturer’s instructions. The severity of elastin degradation was examined via EVG staining and graded as follows: grade 1, no degeneration; grade 2, mild elastin degeneration; grade 3, severe elastin degeneration; and grade 4, aortic rupture^19^.

### Immunohistochemical staining

The aortic tissue sections were deparaffinized, rehydrated, and washed in distilled water. For immunohistochemical analysis, the sections were washed three times with PBS, fixed in 4% PFA for 15 min, and permeabilized with 0.1% Triton X-100 (T9284, Sigma, MO, USA) for 15 min. The sections were incubated with 3% H_2_O_2_ for 10 min and incubated with goat serum (ZLI-9056, ZsBio, Beijing, China) to block endogenous peroxidase activity. Then, the sections were incubated with GPX4 (1:200, ab125066, Abcam, USA) or p-AKT (1:200, 4060S, CST, USA) antibodies overnight at 4 °C. The following day, the sections were washed three times with PBS and incubated with goat anti-rabbit immunoglobulin gamma (IgG) secondary antibodies (ZF-0136, ZsBio, Beijing, China) for 30 min at room temperature. Positively stained cells were visualized by adding 3,3′-diaminobenzidine (GK600505, GeneTech, Shanghai, China).

### Immunofluorescence staining

For immunofluorescence staining, serial sections (5 μm) of paraffin-embedded tissues or primary cultured cells were fixed in 4% PFA for 15 min and permeabilized with 0.1% Triton X-100. The sections were blocked with 1% goat serum at room temperature and incubated with anti-rabbit CitH3 (1:200, ab5103, Abcam, USA), anti-rabbit GPX4 (1:200, ab125066, Abcam, USA), anti-goat TAGLN (SM22α, 1:200, ab10135, Abcam, USA), anti-rabbit cleaved caspase-3 (1:200, ab32042, Abcam, USA), anti-rabbit MPO (1:200, ab208670, Abcam, USA) and anti-rat Ly6G (1:200, ab25377, Abcam, USA) antibodies overnight at 4 °C. For the TdT-mediated dUTP nick-end labeling (TUNEL) assay, the sections were incubated with TUNEL reaction buffer (C1086, Beyotime, China) at 37 °C for 1 h in the dark. The following day, the sections were incubated with fluorescently labeled secondary antibodies diluted in blocking buffer at room temperature for 1 h and mounted with 4′,6-diamidino-2-phenylindole (Vector, ZsBio, Beijing, China). Images were captured using a confocal microscope (Leica-SP8, Wetzlar, Germany).

### NET preparation

Blood samples (5 mL) were collected from all participants via venipuncture and were preserved in EDTA for cell isolation. Initial cell separation was performed using Polymorphprep™ (Axis-Shield PoC, AS), which yielded two well-separated leukocyte fractions: polynuclear cells and monocytes. To obtain cells with increased purity, the polynuclear fraction was sorted based on positive polymorphonuclear cells and suspended in Roswell Park Memorial Institute Medium 1640 supplemented with 5% fetal bovine serum (FBS) and 1% penicillin–streptomycin.

For NET formation, the cells were seeded at a density of 10^6^/well and stimulated with 100 nM phorbol 12-myristate 13-acetate (PMA, Beyotime, China) for 4 h at 37 °C. NET formation was confirmed via the visualization of extracellular DNA stained with SYTOX dye. Then, the medium was carefully removed, and the cell layer was gently washed with 3 mL of PBS without Ca^2+^ or Mg^2+^ ions. The PBS solution was collected after vigorous agitation and centrifuged for 10 min at 500 × *g* and 4 °C to remove residual cells and debris. The concentration of NETs was determined using a Quant-iT PicoGreen dsDNA assay kit (P11496, Thermo Fisher Scientific, Waltham, MA, USA), and NETs were immediately used or stored at −80 °C.

### Cell culture and treatments

Human aortic smooth muscle cells (HASMCs) (Lonza) were cultured in SMCM (ScienCell, CA, USA) supplemented with 10% FBS, 1% smooth muscle growth supplement, and 1% penicillin–streptomycin. HASMCs were serum-starved for 12 h and subsequently treated with the following agents in serum-free SMCM for 8 h at 37 °C and 5% CO_2_: PBS (control group), NETs (NET group), NETs + DNase I (0.1 mg/mL, InvivoGen, San Diego, CA, USA), NETs + 740 Y-P (50 µg/ml, a PI3K/Akt agonist, MedChemExpress, USA), NETs + Fer-1 (1 µM, a ferroptosis inhibitor, MedChemExpress, USA), NETs + Z-VAD-FMK (40 µM, an apoptosis inhibitor, MedChemExpress, USA), and NETs + 3-methyladenine (3-MA, 10 mM, an autophagy inhibitor, MedChemExpress, USA).

### SYTOX Green nucleic acid stain

SYTOX Green is a high-affinity nucleic acid stain that can easily penetrate cells with compromised plasma membranes but will not penetrate the membranes of live cells. It is an excellent DNA counterstain for fixed cells. Neutrophils were washed three times in phosphate-free buffer as required and seeded in a 12-well plate at a density of 5 × 10^4^ cells/well. Then, the cells were incubated with 200 μL of 5 mM SYTOX Green (S7020, Invitrogen, Carlsbad, CA) for 30 min at room temperature in the dark. The staining solution was removed, and the cells were washed three times in phosphate-free buffer. Images were captured on a confocal microscope (Leica-SP8, Wetzlar, Germany). The experiment was performed in triplicate.

### Flow cytometry

An Annexin-V Apoptosis Detection Kit (C1062L, Beyotime, China) was used to quantify the apoptotic rate according to the manufacturer’s instructions. Briefly, the cells were incubated with Annexin V-FITC and propidium iodide in the dark for 30 min, washed twice, and analyzed via flow cytometry (Accuri C6, BD Biosciences). The data were analyzed using FlowJo (version 10) software. The experiment was performed in triplicate, and statistical analysis was performed using GraphPad Prism software.

### Enzyme-linked immunosorbent assay

The concentrations of myeloperoxidase (MPO, ab119605, Abcam, USA) and elastase (DY9167-05, R&D Systems, Minneapolis, MN) in neutrophils were detected using enzyme-linked immunosorbent assay (ELISA) kits according to the manufacturer’s instructions.

### Western blotting

Cells and homogenized tissues were lysed in radioimmunoprecipitation assay buffer, and proteins were separated on 4–20% gradient gels. Protein concentration was measured using the Omni-Easy™ Instant BCA Protein Assay Kit (ZJ102, EpiZyme, China). Primary antibodies against CD9 (1:1000, ab236630), CD63 (1:1000, ab134045), CD81 (1:1000, ab79559), Alix (1:1000, ab275377), calnexin (1:1000, ab133615), CitH3 (1:1000, ab5103), PAD4 (1:1000, ab96758), GPX4 (1:1000, ab125066), ACSL4 (1:1000, ab155282), TFR1 (1:1000, ab214039), and SLC7A11 (1:1000, ab216876) were purchased from Abcam (USA). Primary antibodies against H3 (1:1000, 4499S), phosphoinositide-3-kinase (PI3K) (1:1000, 17366S), p-AKT (1:1000, 4060S), and AKT (1:1000, 4685S) were purchased from Cell Signaling Technology (CST, USA). The anti-GAPDH (housekeeping protein) antibody (1:1000, 5174S) was purchased from CST. Densitometry was performed using ImageJ (version 1.47) software. The experiment was performed in triplicate.

### Statistical analysis

The data are expressed as the mean ± SD or as a percentage. Normality was determined using the Kolmogorov–Smirnov test. Nonnormally distributed variables were log-transformed before statistical analysis. Student’s *t* test was used to compare continuous variables between groups. One-way ANOVA followed by the Student–Newman–Keuls-q (SNK-q) post hoc test was used to compare continuous variables among more than two groups. The chi-squared test was used to compare categorical variables between two groups or among more than two groups. The Gehan–Breslow–Wilcoxon test was used to compare survival curves. Statistical significance was determined at *P* values <0.05, and the data were analyzed using SPSS Statistics (version 22.0) software (SPSS, Chicago, Illinois).

## Results

### Inhibiting NET release protected against AAA formation

The circulating levels of NET markers (CitH3, cfDNA, and nucleosomes) were significantly higher in patients with AAA than in healthy individuals (Supplementary Fig. 2a). Immunoblotting and immunofluorescence staining revealed that the levels of NET markers (CitH3, MPO, and PAD4) were higher in patients with AAA than in healthy individuals (Supplementary Fig. 2b, c). The levels were higher in patients with large (>55 mm), ruptured, and rapidly developing AAAs than in those with small (≤55 mm), unruptured, and slowly developing AAAs (Supplementary Fig. 2d–f). The postoperative circulating levels of NET markers were lower in patients with AAA who received endovascular therapy than in those who received open repair surgery (Supplementary Fig. 2g, h).

Microarray datasets (GSE47472 and GSE98278) from the Gene Expression Omnibus database were used for bioinformatic analysis to examine the expression of NET-related indicators in AAA. PADI4 expression was higher in AAA tissues than in healthy aortic tissues (Supplementary Fig. 3a). High expression of PADI4 was associated with a high risk of rupture and large aneurysm size (Supplementary Fig. 3b).

**Fig. 1:**
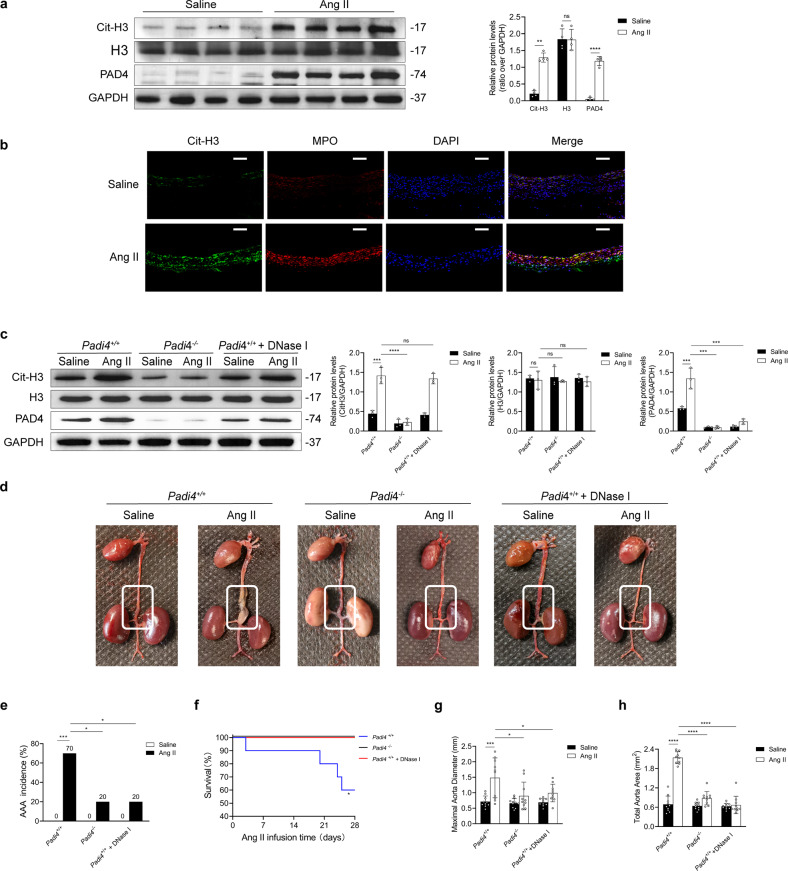
NETs promote AAA formation in the Ang II-induced AAA model. **a** Western blot showing Cit-H3, H3, and PAD4 in the tissue samples of mice injected with Ang II or saline. *n* = 4 in each group. Student’s *t* test. **b** Representative images showing immunofluorescence staining for MPO (red), Cit-H3 (green), and DAPI (blue) in the tissue samples of the AAA mouse model and controls. Scale bar = 150 µm. **c** Western blot showing Cit-H3, H3, and PAD4 in the tissue samples of *ApoE*^−/−^*Padi4*^+/+^, *ApoE*^−/−^*Padi4*^−/−^, and *ApoE*^−/−^*Padi4*^+/+^ + DNase I (10 mL/kg/d)-treated mice infused with saline or Ang II. *n* = 3 in each group, one-way ANOVA followed by the SNK-q post hoc test. **d** Representative macroscopic images of entire aortas harvested from *ApoE*^−/−^*Padi4*^+/+^, *ApoE*^−/−^*Padi4*^−/−^, and *ApoE*^−/−^*Padi4*^+/+^ + DNase I (10 mL/kg/d)-treated mice infused with saline or Ang II. **e** Quantification of aneurysm incidence in the mice. *n* = 10 in each group, Chi-square test. **f** Kaplan–Meier curves showing survival free from aneurysm rupture in 28-day Ang II-infused mice. *n* = 10 in each group, Gehan–Breslow–Wilcoxon test. **g**, **h** Morphometric analysis of the maximal aorta diameter and total aorta area of the mice. *n* = 10 in each group, one-way ANOVA followed by the SNK-q post hoc test. For all subfigures: ns: *P* > 0.05, *P < 0.05, **P < 0.01, ***P < 0.001, ****P < 0.0001, the data are given as the mean ± SD. *Padi4*^+/+^ represents *ApoE*^−/−^*Padi4*^+/+^ mice, *Padi4*^−/−^ represents *ApoE*^−/−^ *Padi4*^−/−^ mice.

Higher expression of NET markers was observed in experimental AAA models than in controls, as assessed by immunoblotting and immunostaining (Fig. 1a, b). The effects of *Padi4* knockout or DNase I treatment on NET formation were verified by Western blotting. *Padi4* knockout or DNase I treatment effectively inhibited NET formation in vivo (Fig. 1c). As shown in Fig. 1d, e, the incidence of Ang II-induced AAA was lower in *ApoE*^−/−^*Padi4*^−/−^ mice and DNase I-treated *ApoE*^−/−^*Padi4*^+/+^ mice than in *ApoE*^−/−^*Padi4*^+/+^ mice. In addition, the survival rate of *ApoE*^−/−^*Padi4*^−/−^ mice and DNase I-treated mice was higher than that of *ApoE*^−/−^*Padi4*^+/+^ mice after Ang II administration (Fig. 1f). Morphometric analysis revealed that the maximal aortic diameter and total aortic area were smaller in Ang II-infused *ApoE*^−/−^*Padi4*^−/−^ mice and DNase I-treated *ApoE*^−/−^*Padi4*^+/+^ mice than in Ang II-infused *ApoE*^−/−^*Padi4*^+/+^mice (Fig. 1g, h).

### NETs promoted AAA formation by inducing ferroptosis in aortic SMCs

Ferroptosis, which is a form of programmed cell death, may be involved in AAA formation^13^. NETs can induce ferroptosis in alveolar epithelial cells during acute lung injury^14^. In this study, we examined the role of NET-mediated ferroptosis in AAA formation. Kyoto Encyclopedia of Genes and Genomes (KEGG) enrichment analysis of AAA-related datasets (GSE98278 and GSE57691) revealed that ferroptosis might play an indispensable role in AAA formation (Supplementary Fig. 4a). The iron-containing enzyme lipoxygenase, whose function relies on the activation of acyl-CoA synthetase long-chain family member 4 (ACSL4), promotes ferroptosis by producing lipid peroxides. In addition, based on the normal expression of cystine/glutamate antiporter solute carrier family 7 member 11 (SLC7A11) and glutathione peroxidase 4 (GPX4), the glutathione-dependent lipid peroxide reduction system plays an important role in the prevention of ferroptosis^20^. Increased expression of the iron importer transferrin receptor 1 (TFR1) can increase iron levels, decrease GSH and GPX4 activity, and enhance lipid peroxidation and the likelihood of ferroptosis^21^. As shown in Supplementary Fig. 4b, the expression of ferroptosis markers (GPX4 and SLC7A11) was lower in AAA tissue samples than in healthy control samples.

**Fig. 2:**
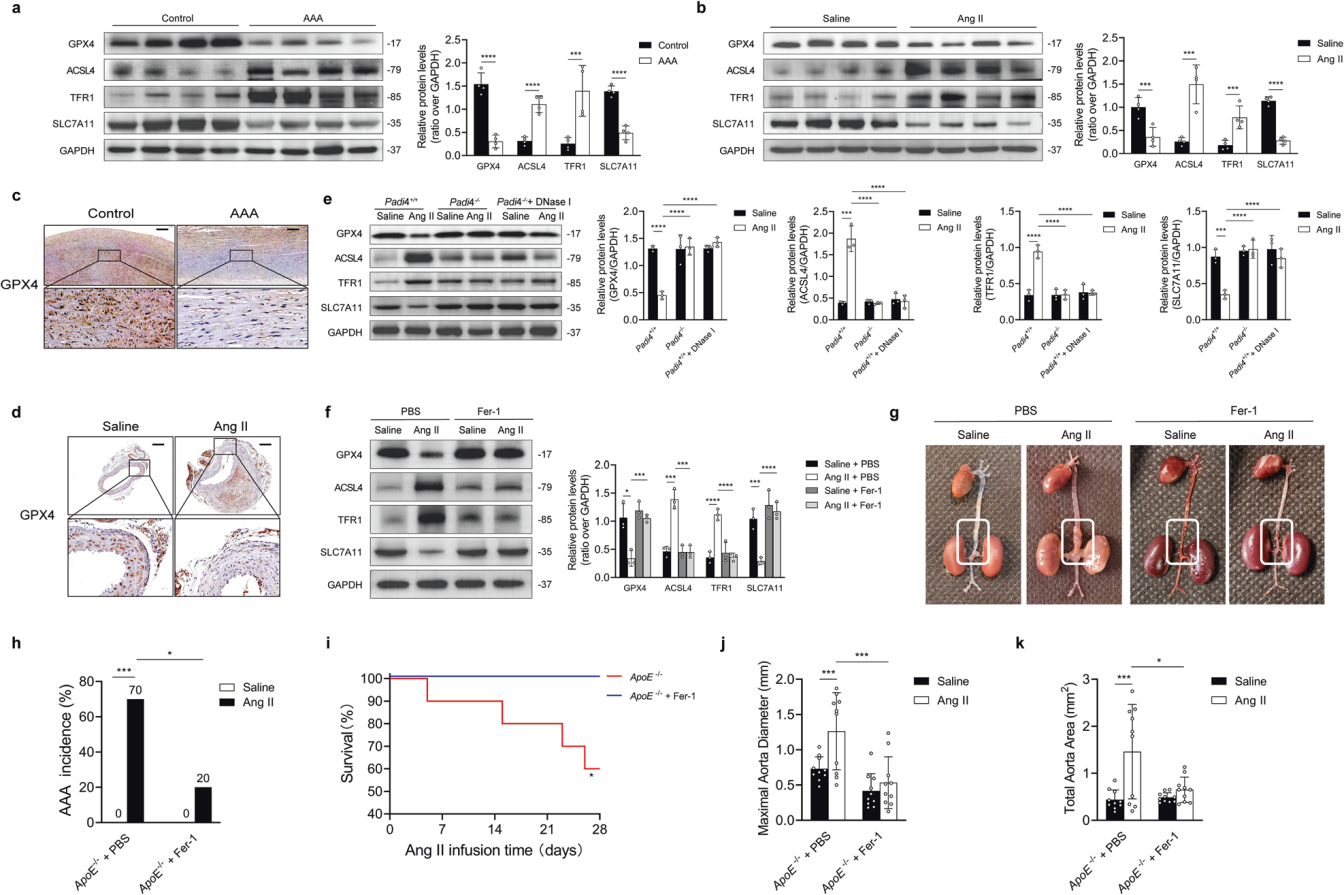
NET-induced ferroptosis promotes AAA formation. **a** Western blot showing GPX4, ACSL4, TFR1, and SLC7A11 in the tissue samples of patients with AAA and controls. *n* = 4 in each group. Student’s *t* test. **b** Western blot showing GPX4, ACSL4, TFR1, and SLC7A11 in the tissue samples of mice injected with Ang II or saline. *n* = 4 in each group. Student’s *t* test. **c** Immunohistochemical staining of GPX4 in tissue samples from patients with AAA and controls. Scale bar = 250 µm. **d** Immunohistochemical staining of GPX4 in tissue samples from the AAA mouse model and controls. Scale bar = 250 µm. **e** Western blot showing GPX4, ACSL4, TFR1, and SLC7A11 in the tissue samples of *ApoE*^−/−^*Padi4*^+/+^, *ApoE*^−/−^*Padi4*^−/−^, and *ApoE*^−/−^*Padi4*^+/+^ + DNase I (10 mL/kg/d)-treated mice infused with saline or Ang II. *n* = 3 in each group, one-way ANOVA followed by the SNK-q post hoc test. **f** Western blot showing GPX4, ACSL4, TFR1, and SLC7A11 in the tissue samples of *ApoE*^−/−^ mice infused with saline or Ang II, with or without Fer-1 (0.8 mg/kg/d) treatment. *n* = 3 in each group, one-way ANOVA followed by the SNK-q post hoc test. **g** Representative macroscopic images of entire aortas harvested from *ApoE*^−/−^ mice infused with saline or Ang II, with or without Fer-1 treatment. **h** Quantification of aneurysm incidence in the mice. *n* = 10 in each group, *χ*^2^ test. **i** Kaplan–Meier curves of survival free from aneurysm rupture in 28-day Ang II-infused mice. *n* = 10 in each group, Gehan–Breslow–Wilcoxon test. **j**, **k** Morphometric analysis of the maximal aorta diameter and total aorta area of the mice. *n* = 10 in each group, one-way ANOVA followed by the SNK-q post hoc test. For all subfigures: *P < 0.05, ***P < 0.001, ****P < 0.0001, the data are given as the mean ± SD. *Padi4*^+/+^ represents *ApoE*^−/−^*Padi4*^+/+^ mice, *Padi4*^−/−^ represents *ApoE*^−/−^ *Padi4*^−/−^ mice.

Western blotting revealed that ferroptosis was upregulated in human and mouse AAA samples compared with control samples (Fig. 2a, b). Consistently, immunohistochemical staining revealed that GPX4 expression was significantly lower in AAA tissues than in healthy tissues (Fig. 2c, d). Inhibiting NET release via *Padi4* knockout or DNase I treatment effectively suppressed ferroptosis in Ang II-infused mice (Fig. 2e). In addition, Fer-1 inhibited Ang II-induced ferroptosis in experimental AAA models (Fig. 2f). As shown in Fig. 2g–i, the incidence of AAA was lower and survival rates were higher in Ang II-infused mice treated with Fer-1 than in Ang II-infused PBS-treated mice. Morphometric analysis revealed that the maximal aortic diameter and total aortic area were smaller in Ang II-infused mice treated with Fer-1 than in Ang II-infused mice treated with PBS (Fig. 2j, k).

Based on the immunohistochemical staining results, the difference in GPX4 expression between AAA and healthy tissues was particularly evident in the aortic media. Given that SMCs are the main cells in the arterial media, we verified SMC ferroptosis by staining for GPX4 and SM22α. Ferroptosis was upregulated in AAA tissues compared with healthy tissues (Supplementary Fig. 5a, b). Western blotting revealed that the expression of ACSL4 and TFR1 was higher and that of GPX4 and SLC7A11 was lower in NET-stimulated SMCs than in control SMCs (Supplementary Fig. 5c). In addition, NET-induced cell death was rescued by DNase I treatment (Supplementary Fig. 5d). Fe^2+^ upregulation, GSH depletion, lipid peroxidation, and ROS accumulation are critical events in ferroptosis^22^. As expected, these events were more pronounced in NET-stimulated SMCs than in PBS-treated SMCs (Supplementary Fig. 5e–h). To assess whether NETs can induce cell death via other mechanisms, Z-VAD-FMK (an apoptosis inhibitor) and 3-MA (an autophagy inhibitor) were used to reverse the effects of NETs on SMCs. As shown in Supplementary Fig. 6a and b, cell viability was not considerably altered after Z-VAD-FMK or 3-MA treatment, which indicated that NETs did not induce SMC death through apoptosis or autophagy. Moreover, we observed that Fer-1 treatment reversed the effect of NETs on cell viability (Supplementary Fig. 6c). The expression of ACSL4 and TFR1 was lower and that of GPX4 and SLC7A11 was higher in NET-stimulated SMCs treated with Fer-1 than in untreated NET-stimulated SMCs (Supplementary Fig. 6d). These results indicated that NET-induced Fe^2+^ accumulation, GSH depletion, lipid peroxidation, and ROS accumulation were reversed by Fer-1 (Supplementary Fig. 6e–h). In addition, NETs reduced the proliferation of SMCs (Supplementary Fig. 7a–c).

### NETs induced SMC ferroptosis by inhibiting the PI3K/AKT pathway

NETosis is associated with the PI3K/AKT signaling pathway, which is downregulated in AAA^23,24^. PI3K/AKT inhibitors are widely used as ferroptosis inducers in immunotherapy^25^. KEGG analysis showed that the PI3K/AKT pathway was closely associated with AAA formation (Supplementary Fig. 8a, b). Bioinformatic tools were used to examine the expression of NET markers, ferroptosis, and activity of the PI3K/AKT pathway in AAA and healthy samples. NETs were strongly correlated with ferroptosis and the PI3K/AKT pathway (Supplementary Fig. 8c, d).

**Fig. 3:**
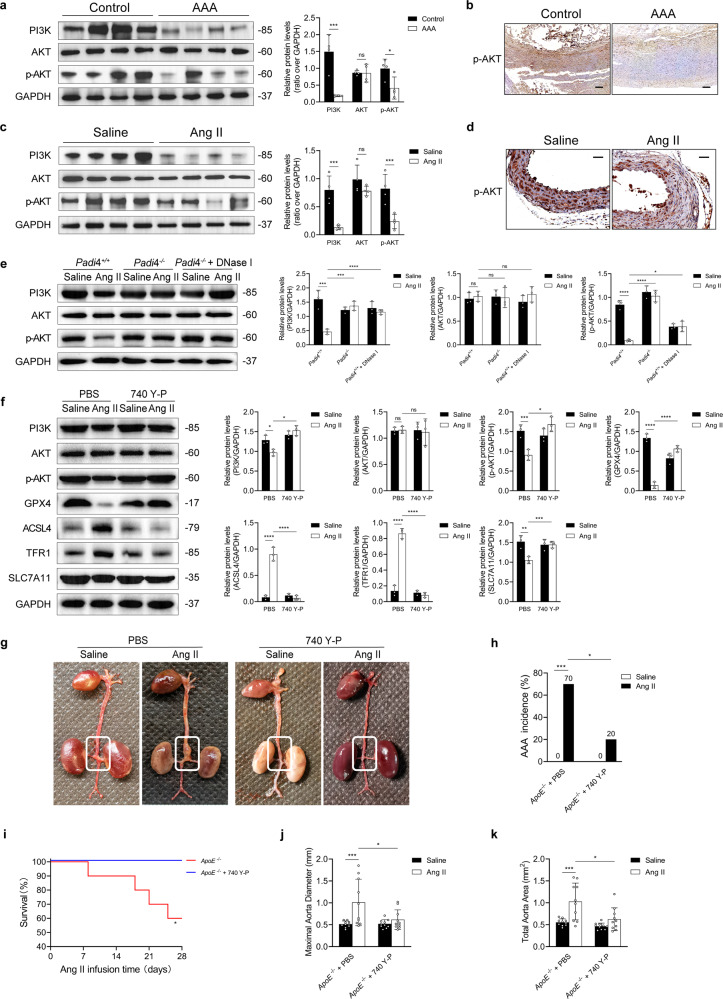
NETs induce ferroptosis by inhibiting the PI3K/AKT pathway. **a** Western blot showing PI3K, AKT, and p-AKT in the tissue samples of patients with AAA and controls. *n* = 4 in each group. Student’s *t* test. **b** Immunohistochemical staining of p-AKT in tissue samples from patients with AAA and controls. Scale bar = 250 µm. **c** Western blot showing PI3K, AKT, and p-AKT in the tissue samples of mice injected with Ang II or saline. *n* = 4 in each group. Student’s *t* test. **d** Immunohistochemical staining of p-AKT in tissue samples from the AAA mouse model and controls. Scale bar = 50 µm. **e** Western blot showing PI3K, AKT, and p-AKT in the tissue samples of *ApoE*^−/−^*Padi4*^+/+^, *ApoE*^−/−^*Padi4*^−/−^, and *ApoE*^−/−^*Padi4*^+/+^ + DNase I (10 mL/kg/d)-treated mice infused with saline or Ang II. *n* = 3 in each group, one-way ANOVA followed by the SNK-q post hoc test. **f** Western blot showing PI3K, AKT, p-AKT, GPX4, ACSL4, TFR1, and SLC7A11 in the tissue samples of *ApoE*^−/−^ mice infused with saline or Ang II, with or without 740 Y-P (10 mg/kg/d) treatment. *n* = 3 in each group, one-way ANOVA followed by the SNK-q post hoc test. **g** Representative macroscopic images of entire aortas harvested from *ApoE*^−/−^ mice infused with saline or Ang II, with or without 740 Y-P treatment. **h** Quantification of aneurysm incidence in the mice. *n* = 10 in each group, Chi-square test. **i** Kaplan–Meier curves of survival free from aneurysm rupture in 28-day Ang II-infused mice. *n* = 10 in each group, Gehan–Breslow–Wilcoxon test. **j**, **k** Morphometric analysis of the maximal aorta diameter and total aorta area of the mice. *n* = 10 in each group, one-way ANOVA followed by the SNK-q post hoc test. For all subfigures: ns: *P* > 0.05, *P < 0.05, **P < 0.01, ***P < 0.001, ****P < 0.0001, the data are given as the mean ± SD. *Padi4*^+/+^ represents *ApoE*^−/−^*Padi4*^+/+^ mice, *Padi4*^−/−^ represents *ApoE*^−/−^ *Padi4*^−/−^ mice.

PI3K/AKT signaling was significantly suppressed in human and murine AAA samples (Fig. 3a–d). Furthermore, in vivo experiments demonstrated that inhibiting NET formation via *Padi4* knockout or DNase I treatment effectively reversed the downregulation of the PI3K/AKT pathway (Fig. 3e). Subsequently, the effects of the PI3K activator 740 Y-P on ferroptosis were examined. 740 Y-P reversed ferroptosis and upregulated the PI3K/AKT pathway in mice with Ang II-induced AAA (Fig. 3f). As shown in Fig. 3g–i, the incidence of AAA was lower and survival rates were higher in Ang II-infused mice treated with 740 Y-P than in Ang II-infused mice treated with PBS. Morphometric analysis revealed that the maximal aortic diameter and total aortic area were significantly smaller in Ang II-infused mice treated with 740 Y-P than in Ang II-infused mice treated with PBS (Fig. 3j, k).

In vitro experiments demonstrated that NETs inhibited the PI3K/AKT signaling pathway in SMCs (Supplementary Fig. 9a). The expression of ACSL4 and TFR1 was lower and that of GPX4 and SLC7A11 was higher in NET-stimulated SMCs treated with 740 Y-P than in NET-stimulated SMCs (Supplementary Fig. 9b). In addition, 740 Y-P reversed NET-induced cell death, Fe^2+^ upregulation, GSH depletion, lipid peroxidation and ROS accumulation (Supplementary Fig. 9c–g). These results suggest that NETs induce SMC ferroptosis by inhibiting the PI3K/AKT pathway.

### MSC-EVs attenuated AAA formation in Ang II-infused mice

MSC-EVs were isolated from the supernatants of MSCs and characterized by transmission electron microscopy (TEM), dynamic light scattering (DLS), and immunoblotting. TEM revealed the classic sphere-shaped morphology of MSC-EVs (Supplementary Fig. 10a). Particle size distribution by DLS showed that the majority of MSC-EVs were 50–220 nm in diameter, and the most abundant particle size was 125.2 ± 36.50 nm (Supplementary Fig. 10b). Immunoblotting revealed that EV-associated protein markers (CD9, CD63, CD81, and Alix) were enriched in MSC-EVs compared with MSC lysates, whereas the endoplasmic reticulum marker calnexin (a negative protein marker) was not found in MSC-EVs (Supplementary Fig. 10c).

**Fig. 4:**
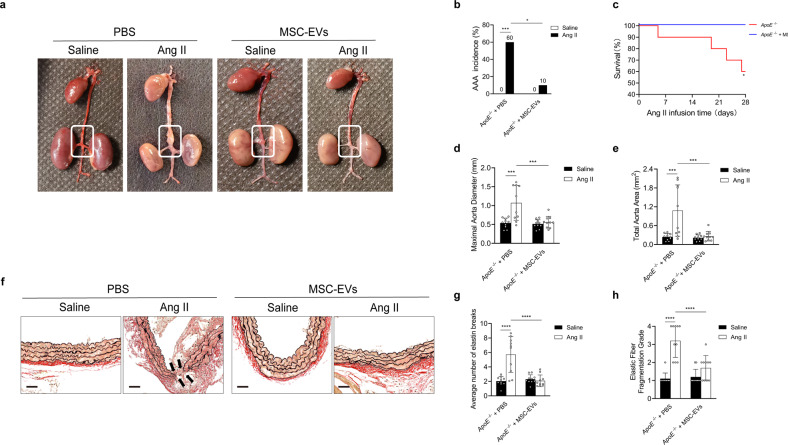
MSC-EVs have therapeutic effects on AAA formation. **a** Representative macroscopic images of entire aortas harvested from *ApoE*^−/−^ mice infused with saline or Ang II with or without MSC-EV (7 × 10^10^ particles/mouse) treatment. **b** Quantification of aneurysm incidence in the mice. *n* = 10 in each group, Chi-square test. **c** Kaplan–Meier curves of survival free from aneurysm rupture in 28-day Ang II-infused mice. *n* = 10 in each group, Gehan–Breslow–Wilcoxon test. **d**, **e** Morphometric analysis of the maximal aorta diameter and total aorta area of the mice. *n* = 10 in each group, one-way ANOVA followed by the SNK-q post hoc test. **f** Representative images showing EVG staining of the abdominal aorta samples of mice. Scale bar = 100 µm. **g**, **h** Quantification of the average number of elastin breaks per vessel and the elastic fiber fragmentation grade of the mice. *n* = 10 in each group, one-way ANOVA followed by the SNK-q post hoc test. For all subfigures: *P < 0.05, ***P < 0.001, ****P < 0.0001, the data are given as the mean ± SD.

AAA formation was reduced in Ang II-infused mice treated with MSC-EVs (AAA was defined as an aortic diameter ≥1.5 times the mean aortic diameter of saline-infused mice) (Fig. 4a). The incidence of AAA formation was lower and survival rates were higher in Ang II-infused mice treated with MSC-EVs than in untreated Ang II-infused mice (Fig. 4b, c). Morphometric analysis revealed that the maximal aortic diameter and total aortic area were significantly smaller in Ang II-infused mice treated with MSC-EVs than in untreated Ang II-infused mice (Fig. 4d, e). Histological analysis revealed degeneration of the aortic media in the aorta walls, which was accompanied by elastin degradation, as evidenced by the widened space between the elastic lamina in untreated Ang II-infused mice. These histological features of aneurysms were significantly attenuated in Ang II-infused mice treated with MSC-EVs compared control mice (Fig. 4f). The average number of breaks was larger and elastin fragmentation was more severe in PBS-treated mice than in MSC-EV-treated mice after 4 weeks of Ang II infusion (Fig. 4g, h).

### MSC-EVs inhibited NET-induced ferroptosis and the downregulation of the PI3K/AKT pathway in the Ang II-infused model

**Fig. 5:**
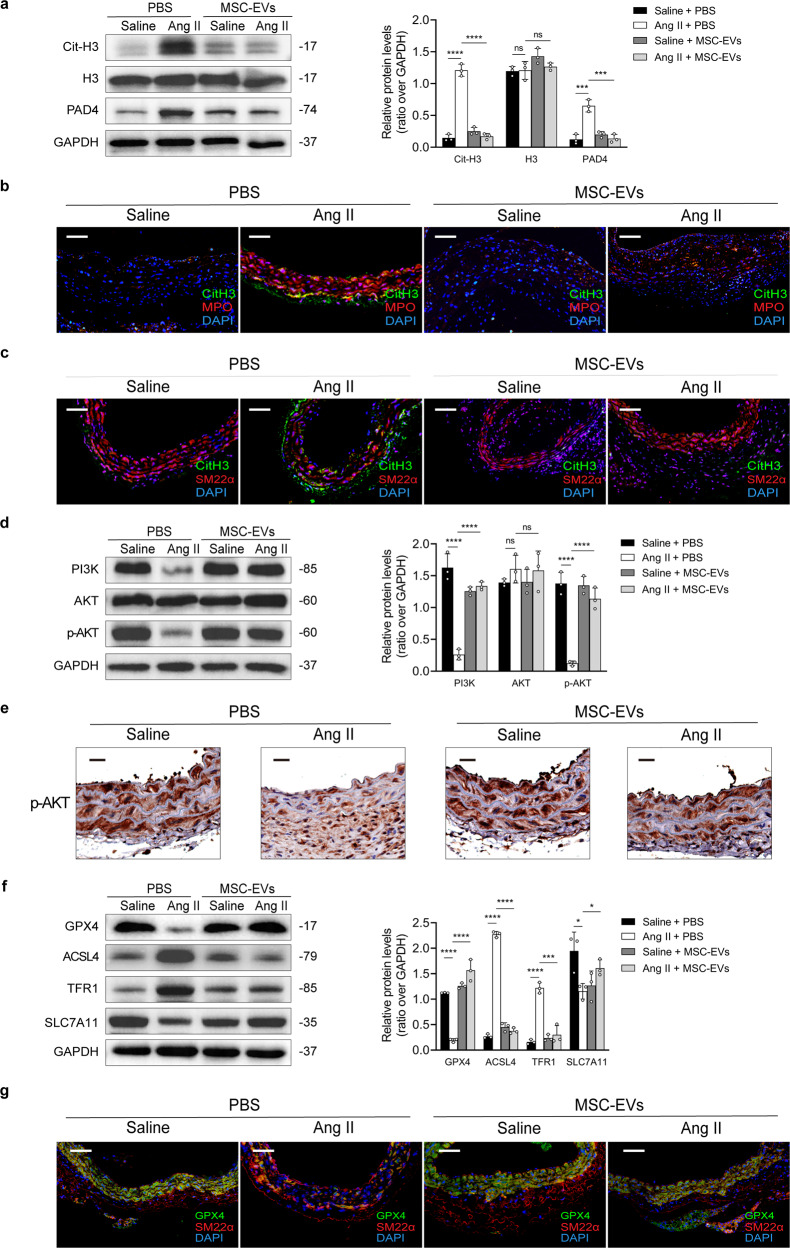
MSC-EVs inhibit NET-induced ferroptosis and suppress the PI3K/AKT pathway. **a** Western blot showing Cit-H3, H3, and PAD4 in the tissue samples of *ApoE*^−/−^ mice infused with saline or Ang II, with or without MSC-EV (7 × 10^10^ particles/mouse) treatment. **b** Representative images showing immunofluorescence staining for MPO (red), Cit-H3 (green), and DAPI (blue) in the aortic samples of mice. Scale bar = 150 µm. **c** Representative images showing immunofluorescence staining for SM22α (red), Cit-H3 (green), and DAPI (blue) in the aortic samples of mice. Scale bar = 150 µm. **d** Western blot showing PI3K, AKT, and p-AKT in the tissue samples of *ApoE*^−/−^ mice infused with saline or Ang II, with or without MSC-EV (7 × 10^10^ particles/mouse) treatment. **e** Immunohistochemical staining of p-AKT in tissue samples from the AAA mouse model and controls. Scale bar = 50 µm. **f** Western blot showing GPX4, ACSL4, TFR1, and SLC7A11 in the tissue samples of *ApoE*^−/−^ mice infused with saline or Ang II, with or without MSC-EV (7 × 10^10^ particles/mouse) treatment. **g** Representative images showing immunofluorescence staining for SM22α (red), GPX4 (green), and DAPI (blue) in the aortic samples of mice. Scale bar = 150 µm. For all subfigures: ns: *P* > 0.05, *P < 0.05, ***P < 0.001, ****P < 0.0001, the data are given as the mean ± SD. *n* = 3 in each group, one-way ANOVA followed by the SNK-q post hoc test.

Several in vivo experiments were performed to investigate the effects of MSC-EVs on NET-mediated ferroptosis during AAA formation. MSC-EVs downregulated the expression of NET markers (CitH3, MPO, and PAD4) in Ang II-induced mice (Fig. 5a, b). Immunofluorescence staining revealed that MSC-EVs significantly reduced the accumulation of NETs around SMCs (Fig. 5c). MSC-EVs reversed the downregulation of the PI3K/AKT signaling pathway in the Ang II-infused mouse model (Fig. 5d, e). Western blotting showed that the expression of ACSL4 and TFR1 was lower and that of GPX4 and SLC7A11 was higher in MSC-EV-treated mice with AAA than in control mice (Fig. 5f). In addition, MSC-EVs effectively reduced GPX4 expression in SMCs in AAA mouse models (Fig. 5g).

### MSC-EVs redirected NETosis to apoptosis

NETs significantly reduced the proliferation of SMCs, and these changes could not be reversed by MSC-EVs (Supplementary Fig. 11a–c). In addition, we found that NETs and MSC-EVs could not affect the migration of SMCs (Supplementary Fig. 11d). Given that MSC-EVs did not reverse NET-induced ferroptosis and the downregulation of the PI3K/AKT pathway in SMCs (Supplementary Fig. 11e, f), we hypothesized that MSC-EVs attenuated ferroptosis and the downregulation of the PI3K/AKT pathway by inhibiting NET formation in vivo.

**Fig. 6:**
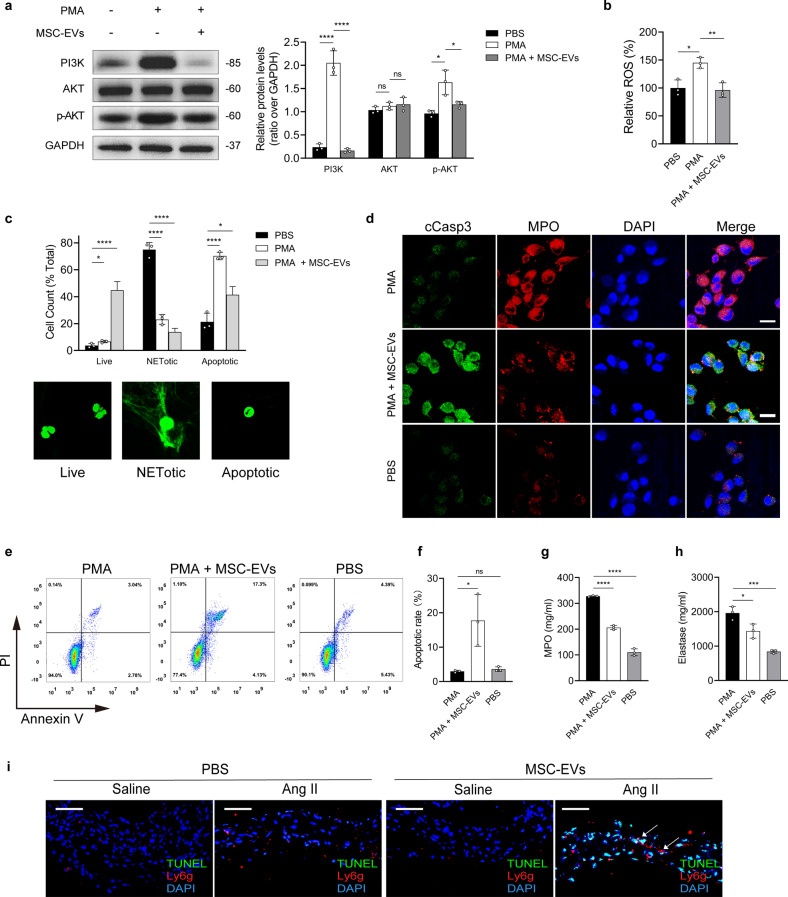
MSC-EVs redirect NETosis to apoptosis in neutrophils. **a** Western blot showing PI3K, AKT, and p-AKT in neutrophils treated with PMA (100 nM) for 4 h, with or without MSC-EV (200 ng/μL) treatment. *n* = 3 in each group, one-way ANOVA followed by the SNK-q post hoc test. **b** The relative levels of lipid ROS in neutrophils were assayed. *n* = 3 in each group, one-way ANOVA followed by the SNK-q post hoc test. **c** Differential quantification of live, NETotic, and apoptotic (pyknotic) nuclei in neutrophils. *n* = 3 in each group, one-way ANOVA followed by the SNK-q post hoc test. **d** Immunofluorescence staining for MPO (NET marker) and cCasp3 (apoptosis marker). Scale bar = 10 µm. **e**, **f** Annexin V/PI staining in the groups was analyzed by flow cytometry. *n* = 3 in each group, one-way ANOVA followed by the SNK-q post hoc test. **g**, **h** The concentrations of MPO and elastase were tested by ELISA. *n* = 3 in each group, one-way ANOVA followed by the SNK-q post hoc test. **i** Representative images showing immunofluorescence staining for Ly6G (red), TUNEL (green), and DAPI (blue) in the aortic samples of mice. Scale bar = 150 µm. For all subfigures: ns: *P* > 0.05, *P < 0.05, ***P < 0.001, ****P < 0.0001, the data are given as the mean ± SD.

MSC-derived apoptotic vesicles can inhibit NET formation by redirecting NETosis to apoptosis^26^. In this study, we examined whether MSC-EVs inhibited NET formation by regulating the NETosis–apoptosis axis. It has been reported that PMA activates AKT, a well-known inhibitor of apoptosis, and that AKT is a direct molecular switch that regulates the NETosis–apoptosis axis^27^. PMA has been extensively used as an agonist to promote ROS production to study NETosis, and ROS production by neutrophils plays a critical role in AKT activation and NETosis^27^. Phosphorylation is an important manifestation of AKT activation, which can be observed by immunoblotting. MSC-EV pretreatment effectively reversed PMA-induced AKT activation and ROS production (Fig. 6a, b). Immunofluorescence microscopy and quantitative analysis showed that MSC-EVs increased the number of apoptotic cells containing pyknotic nuclei in the PMA-stimulated group but decreased the number of NETotic cells (Fig. 6c). Immunofluorescence staining of MPO (a NETosis marker) and cleaved caspase 3 (cCasp3, an apoptosis marker) revealed that MSC-EVs redirected PMA-induced NETosis to apoptosis in neutrophils (Fig. 6d). Flow cytometry showed a significant increase in the proportion of apoptotic neutrophils after MSC-EV treatment (Fig. 6e, f). The concentrations of MPO and elastase in neutrophils were evaluated by ELISA, and the results indicated that MSC-EVs inhibited PMA-stimulated NETosis (Fig. 6g, h). The proportion of apoptotic neutrophils, which were identified as cells that were positively stained for TUNEL, Ly6G, and DAPI, was significantly higher in MSC-EV-treated mice with Ang II-induced AAA than in PBS-treated mice (Fig. 6i). Immunofluorescence staining, SYTOX green staining, and flow cytometry were used to quantitively confirm the levels of NETosis and apoptosis in neutrophils in AAA models. Immunofluorescence and SYTOX green staining showed that MSC-EVs effectively reduced the levels of NET markers and NET fibers in the aortic tissues of AAA model mice (Supplementary Fig. 12a, b). Moreover, MSC-EV treatment increased the level of Ly6G^+^TUNEL^+^ cells (apoptotic neutrophils) in the aortic tissues of Ang II-induced AAA model mice (Supplementary Fig. 12c, d).

To investigate how MSC-EVs are internalized by neutrophils, the respective inhibitors, including EIPA (an inhibitor of micropinocytosis), CPZ (an inhibitor of clathrin-mediated endocytosis), and nystatin (an inhibitor of caveolae-mediated endocytosis), were used to examine the endocytic pathway through which MSC-EVs are internalized. MSC-EVs were labeled with PKH26. An internalization assay showed that MSC-EVs were internalized via the clathrin-mediated endocytic pathway (Supplementary Fig. 13a). These results suggest that MSC-EVs act by inhibiting NET formation instead of regulating the effects of NETs on ferroptosis in AAA mouse models.

## Discussion

**Fig. 7:**
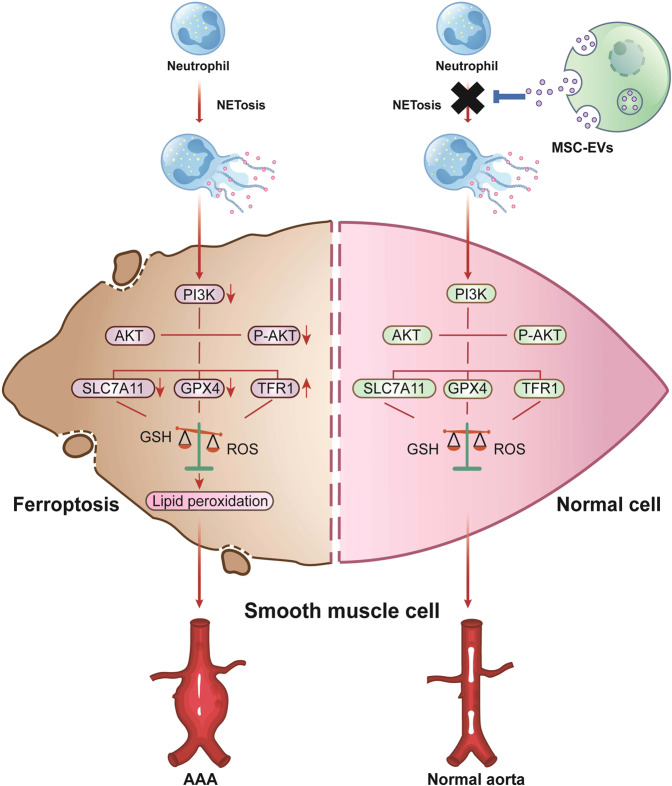
MSC-EVs protect against AAA formation by inhibiting NET-induced SMC ferroptosis. NETs promote AAA formation by inducing SMC ferroptosis by inhibiting the PI3K/AKT pathway, and MSC-EVs can protect against AAA formation by inhibiting NETosis in Ang II-induced AAA models.

This study demonstrated that NETs promote AAA formation by inducing SMC ferroptosis by inhibiting the PI3K/AKT pathway, and MSC-EVs can protect against AAA formation by inhibiting NETosis in Ang II-induced AAA models (Fig. 7). Furthermore, our results revealed that MSC-EVs inhibited NET formation by redirecting NETosis to apoptosis in neutrophils. These results suggest a potential pharmacological role of MSC-EVs in AAA treatment.

Neutrophils function in a variety of ways, and NETs are prominent in AAA formation^8^. An increase in NET markers was observed in the plasma of AAA patients compared with that of healthy subjects^28^. NET components are circulating biomarkers for predicting AAA progression^29^. Consistently, in this study, we found that NET components were enriched in the serum and aortic tissues of patients with AAA, and the levels of NETs were valuable for predicting the size, rate of progression, and risk of rupture of AAA. In addition, postoperative circulating levels of NET markers were decreased in patients with AAA who received endovascular therapy but not in those who received open repair surgery, which may be attributed to the wide release of NETs triggered during open repair surgery^30^.

SMC depletion and dysfunction are major pathological characteristics of AAA^10^. In our previous study, we found that NETs contributed to AAA formation by promoting the synthetic and proinflammatory phenotype in SMCs^31^. Recently, SMC ferroptosis has been shown to be closely associated with various types of vascular diseases, such as vascular calcification, aortic dissection, and neointimal hyperplasia^32–34^. This study is the first to report that ferroptosis is a regulated cell death type in SMCs during AAA formation. Furthermore, we found that ferroptosis-inhibiting agents significantly attenuated AAA formation in vivo and may serve as potential therapeutic agents for AAA treatment.

NETs can induce ferroptosis in a variety of cells. For instance, NETs can mediate N6-methyladenosine modification and regulate sepsis-associated acute lung injury by activating ferroptosis in alveolar epithelial cells^14^. Furthermore, myeloperoxidase, a neutrophil-specific granule and the main component of NETs, can directly induce ferroptosis in glioblastoma cells^35^. However, the underlying mechanism of NET-induced SMC ferroptosis in AAA remains unclear, and further investigation is required to identify the specific NET components and intracellular signal transduction processes that induce SMC ferroptosis during AAA formation.

Considering that PI3K is a significant mediator of ferroptosis, PI3K inhibitors are promising therapeutic agents to induce immunogenic ferroptosis and potentiate cancer immune checkpoint therapy^36^. Our results suggested that NETs induced SMC ferroptosis by inhibiting the PI3K/AKT pathway and that the PI3K agonist 740 Y-P can inhibit SMC ferroptosis and attenuate AAA formation in an Ang II-infused AAA mouse model. The PI3K family, which plays an important role in regulating various cellular functions during inflammatory diseases, has been proposed as a potential target of immunotherapy for AAA^37^. In addition, gamma delta T (γδT) cell deficiency protects against AAA formation by regulating the PI3K/AKT pathway^38^. Further studies may focus on the role of each PI3K isoform at each stage of the AAA immune-inflammatory response for a deeper understanding of the mechanism of inflammation and immune response during AAA formation.

MSCs are effective therapeutic agents for AAA due to their regenerative and immunomodulatory abilities^15^. MSCs have been reported to ameliorate inflammation-associated diseases, including acute lung injury and ocular chemical burns, by inhibiting NET formation^39,40^. In this study, we investigated the role of MSC-EVs in NET release during AAA formation. We observed that tail vein injection of MSC-EVs significantly reduced NET formation, elastin degeneration, and arterial dilatation in the Ang II-induced AAA model. Recently, MSC-derived exosomes were shown to exert therapeutic effects by suppressing ferroptosis in acute liver injury^41^. Our data showed that MSC-EVs significantly inhibited NET-induced ferroptosis in vivo but were unable to inhibit NET-induced ferroptosis in SMCs in vitro. Therefore, instead of directly affecting SMC ferroptosis, MSC-EVs may contribute to the prevention of NETosis, thus protecting against AAA formation.

NETosis and apoptosis are opposing pathways of cellular death in neutrophils, and switching from NETosis to apoptosis could provide avenues for novel therapeutic strategies to treat NET-associated inflammatory disorders^27^. MSC-derived apoptotic vesicles have been shown to improve the survival rates of septic mice by redirecting neutrophil cell death from NETosis to apoptosis^26^. PMA leads to significant increases in NET formation, ROS production, DNA release, and elastase accumulation in neutrophils^42^. PMA was used as an agonist to activate ROS production to explore NETosis in this study. We observed that PMA-induced NETosis was inhibited by MSC-EV treatment, as indicated by the suppression of ROS, MPO, and elastase levels. Moreover, MSC-EV treatment was shown to induce caspase-dependent apoptosis in PMA-stimulated neutrophils. Therefore, MSC-EVs ameliorated the NET burden, thus acting as promising pharmacological agents for the treatment of various diseases characterized by excessive NET release, such as AAA.

This study has several limitations. First, since the characteristics of Ang II-induced AAA are consistent with activation of the inflammatory response and stimulation of the protein hydrolysis cascade reaction, we used Ang II-infused *ApoE*^−/−^ mice, which is a widely used AAA model, in this study. Our findings need to be confirmed in other AAA models, such as elastase perfusion-induced and CaCl_2_-induced AAA models. Second, MSC-EVs contain various biological components, including mRNAs, ncRNAs, and proteins, which may be involved in the immunomodulatory effects on NET formation, thus warranting further research to determine the specific components involved in NET formation. Third, although our previous study demonstrated that TLR-9 mediated NET-induced Hippo pathway suppression, the receptors that mediate NET-induced PI3K/AKT pathway inactivation in SMCs are still unknown^31^.

In conclusion, NETs can induce AAA formation by promoting SMC ferroptosis by inhibiting the PI3K/AKT pathway. In contrast, MSC-EVs can inhibit NET formation by redirecting NETosis to apoptosis in neutrophils. This study provides several potential targets for developing novel pharmacological modalities for AAA, and one such treatment strategy for AAA can be via MSC-EVs.

## Supplementary information

Supplemental material clean copy
