# Supplementary material for: Mesenchymal stem cell-derived extracellular vesicles protect against abdominal aortic aneurysm formation by inhibiting NET-induced ferroptosis
Source: Exp Mol Med. 2023 May 1;55(5):939–51. doi: 10.1038/s12276-023-00986-2 (PMC10238484; doi:10.1038/s12276-023-00986-2)
Supplement: Supplementary file 1 — Supplemental material clean copy [file 12276_2023_986_MOESM1_ESM.pdf]

Markers of NETs

Citrullinated H3 (CitH3) was determined according to the manufacturer's instructions. Plasma samples were mixed with a monoclonal mouse anti-histone biotinylated antibody (SEW010106, Kemin Biotechnology Co., Ltd., Shanghai, China) in a streptavidin-coated plate. The second stage uses rabbit histone H3 (ab176842, Abcam) antibody. The peroxidase-linked antibody (MBS135301, DakoCytomation) was used to detect histone H3 antibody. Values were normalized to the sample pool of healthy controls included in all microplates. Values are expressed as individual absorption values. Cell-free double-stranded DNA (cfDNA) was measured after phenol extraction using a Qubit 2.0 fluorometer (Thermo Fisher Scientific, Courtaboeuf, France). Nucleosomes were measured with the Cell Death Detection ELISA^{PLUS} kit (Roche, Germany) according to the manufacturer's instructions.

NET preparation

Blood samples (5 mL) were collected from all participants via venipuncture and preserved in EDTA for cell isolation. Initial cell separation was performed using Polymorphprep™ (Axis-Shield PoC, AS), yielding two well-separated leukocyte fractions: polynuclear cells and monocytes. To obtain cells with higher purity, the polynuclear fraction was sorted based on positive polymorphonuclear cells and suspended in the Roswell Park Memorial Institute Medium 1640 medium supplemented with 5% foetal bovine serum (FBS) and 1% penicillin–streptomycin.

For NET formation, the cells were seeded at a density of 10^6/well and stimulated

with 100nM phorbol 12-myristate 13-acetate (PMA, Beyotime, China) for 4 h at 37°C. NET formation was confirmed via visualisation of extracellular DNA stained with SYTOX dye. Thereafter, the medium was carefully removed, and the cell layer was gently washed with 3 mL of PBS without Ca^{2+} and Mg^{2+} ions. The PBS solution was collected after vigorous agitation and centrifuged for 10 min at 500 g and 4°C to remove residual cells and debris. The concentration of NETs was determined using the Quant-iT PicoGreen dsDNA assay kit (P11496, Thermo Fisher Scientific, Waltham, MA, USA), and NETs were immediately used or stored at -80°C.

Enzyme-linked immunosorbent assay

The concentration of myeloperoxidase (MPO, ab119605, Abcam, USA) and elastase (DY9167-05, R&D Systems, Minneapolis, MN) in neutrophils was detected using enzyme-linked immunosorbent assay (ELISA) kits according to the manufacturer's instructions.

Data collection and analysis

The microarray data of AAA were downloaded from the National Center for Biotechnology Information Gene Expression Omnibus (NCBI_GEO) database. The species type as "Homo sapiens." was set as a filter and the results obtained included three datasets GSE47472, GSE57691, GSE98278, and GSE7084. Each dataset includes data from patients with AAA and normal aorta (which will be referred to as the healthy control group). The differentially expressed mRNAs (DEmRNAs) between the AAA patients and the healthy control group were analyzed and compared using the "Linear Models for Microarray Data (limma)" R package. mRNAs were

considered as DEmRNAs if they met the criteria: |log2 fold change (FC)| > 1 and false discovery rate (FDR) adjusted p-value < 0.05.

Kyoto Encyclopedia of Genes and Genomes (KEGG) enrichment analysis were used to explore the biological functions of DEmRNAs in AAA. The KEGG enrichment analysis was performed using the "clusterProfiler" package of the R software. FDR adjusted p-value < 0.05 was considered statistically significant. Spearman's rank correlation analysis was performed to study the correlation between the target genes. p-value < 0.05 was accepted as statistically significant.

Cell viability assay

For the cell viability assay, cells were evaluated with Cell Counting Kit 8 (Dojindo Molecular Technologies, Rockville, MD, USA) as per the manufacturer's protocol. SMCs were seeded in a 96-well plate at 5×10^3 cells/well in triplicate. The cells were cultured in a humidified atmosphere of 95% air and 5% CO_2 at 37 °C. The absorbance was read at 450 nm using a microplate reader (ThermoFisher, USA).

Ferroptosis marker

Fe^{2+}. The relative iron concentration in cell lysates was assessed with an Iron Assay Kit (ab83366; Abcam) according to the manufacturer's instructions.

GSH/GSSG. Total GSH and glutathione disulfide (oxidized glutathione, GSSG) were evaluated by a GSSG/GSH quantification kit (No. G263; Dojindo, Kumamoto, Japan), according to the manufacturer's instructions. The amount of reduced GSH (shown as GSH) was calculated as total glutathione – 2GSSG, and the ratio of GSH to GSSG was shown.

Lipid Peroxidation. MDA is a major indicator of lipid peroxidation, which was assessed with a Lipid Peroxidation assay kit (ab118970, Abcam, USA) according to the manufacturer's instructions.

Reactive oxygen species (ROS). The relative ROS level in cell lysates was measured by the ROS Assay Kit (S0033M, Beyotime, China) according to the manufacturer's instructions.

Cell proliferation assays

For cell proliferation assays, cells were evaluated with Cell Counting Kit 8 (Dojindo Molecular Technologies, Rockville, MD, USA) according to the manufacturer's protocol. SMC were seeded in a 96-well plate at 5×10^3 cells/well in triplicate. The cells were cultured in a humidified atmosphere of 95% air and 5% CO_2 at 37 °C. The absorbance was read at 450 nm using a microplate reader (ThermoFisher, USA).

For the 5-ethynyl-2′-deoxyuridine (EdU) assay, cells in logarithmic growth phase were seeded in 24-well plates and the appropriate concentration of EDU reagent was added for 4 h. Cell proliferation was measured using the BeyoClick EdU Cell Proliferation Kit with Alexa Fluor 488 (Beyotime, China) according to the manufacturer's protocol. The proliferative cells after EdU incorporation produced an intense uniform green fluorescence. Cell nuclei were stained with Hoechst 33342 dye, which emits bright blue fluorescence.

Cell migration assay

Cell migration was assessed with Transwell Permeable Supports (24-well, 3.0-

μm membrane, Corning Inc., Corning, NY, USA). SMC (5×10^4 cells/mL) were seeded in the upper chamber with serum-free SMCM, while SMCM contain 10% FBS was added in the lower chamber. After incubation at 37°C and 5% CO2 for 24 h, cells migrating to the lower chamber were fixed with 4% PFA, stained with crystal violet solution (Beyotime, China) and counted under a light microscope.

MSC-EVs isolation and identification

The procedures for isolating MSC-EVs were approved by the Institutional Review Board of the Shanghai Jiaotong University School of Medicine, Renji Hospital. MSC-EVs were isolated from the supernatants of human umbilical cord derived MSC (hUC-MSC). The supernatants were obtained from Biotherapy Department of the Renji Hospital, Shanghai Jiaotong University School of Medicine. Supernatant was centrifuged at 3000 g (10 min, 4℃), and 10,000 g (10 min, 4℃). The cellular debris was discarded and the supernatants were transferred to ultracentrifuge tubes. MSC-EVs were purified through two ultracentrifugation steps at 100,000 g (2 h, 4℃) and then resupended in PBS. The morphology of MSC-EVs were observed by transmission electron microscopy (TEM, JEM-1200EX, JEOL, Japan), and the particle size distribution was detected by dynamic light scattering (DLS, Litesizer 500, Anton Paar, Austria). EV-associated proteins, such as CD9, CD63, CD81, and Alix are detected by Western blotting.

SYTOX Green nucleic acid stain

For analysis of NETs *in vivo*, the aorta tissue sections were incubated with SYTOX green (5 mM) and the section was prepared for confocal microscopy. For

each mouse, SYTOX green positive single NET fibers were counted in the aortic tissue and averaged. Data is presented as the average number of fibers of tissue.

Flow cytometry

The cells isolated from abdominal aorta were sorted by flow cytometry to obtain neutrophils using anti-rat Ly6G (1:200, ab25377, Abcam, USA). The Annexin-V Apoptosis Detection Kit (C1062L, Beyotime, China) was used to quantify the apoptotic rate according to the manufacturer's instructions. Briefly, cells were incubated with Annexin V-FITC and propidium iodide in the dark for 30 min, washed twice and analysed via flow cytometry (Accuri C6, BD Biosciences). Data were analysed using the FlowJo (version 10) software. The experiment was performed in triplicate, and statistical analysis was performed using the GraphPad Prism software.

Exosomes internalization assay

MSC-EVs were labelled with PKH26 Cell Membrane Labelling Dye (10^{-6} M, PKH26GL, Sigma-Aldrich, St. Louis, MO, USA) to investigate the uptake of MSC-EVs by neutrophils. Briefly, after incubation with PKH26 for 1 h at 37 °C, PKH26-labelled MSC-EVs were collected by ultracentrifugation at 100,000 g for 2 h at 4 °C and then quenched with PBS containing 10% BSA. Neutrophils obtained from the peripheral blood of healthy donors were incubated with PKH26-labeled MSC-EVs for 2, 4, and 6 h at 37°C. Cells were then fixed with 4% PFA for 30 min at room temperature before nuclei were stained with DAPI for 10 min in the dark. Cell imaging was observed by confocal microscope (Leica-SP8, Wetzlar, Germany).

To investigate the mechanism of MSC-EVs internalization by neutrophils, we

applied various endocytic inhibitors, including chlorpromazine (CPZ, 20μM, HY-12708, MedChemExpress, USA), EIPA (50μM, HY-101840, MedChemExpress, USA) and nystatin (20μM, HY-17409, MedChemExpress, USA).

Supplementary Table 1. Demographics and Baseline Characteristics

Characteristics	Controls (N=51)	AAA patients (N= 22)	*P*-value
**Age, mean ± SD (rang), y**	60.1 ± 11.4 (40-90)	61.0 ± 11.3 (40-78)	0.75
**Gender, n (%)**			
Female	19 (37.3)	7 (31.8)	0.66
Male	32 (62.7)	15 (68.2)	
**Medical history and risk factors, n (%)**			
Smoker status			
Never	22 (43.1)	4 (18.2)	<0.05
Past	10 (19.6)	5 (22.7)	
Current	14 (27.5)	12 (54.5)	
Unknown	5 (9.8)	1 (4.5)	
Hypertension	29 (56.9)	15 (68.2)	0.91
Hyperlipidemia	31 (60.8)	12 (54.5)	0.50
Diabetes Mellitus	21 (41.2)	7 (31.8)	0.75
Peripheral artery disease			
No	25 (49.0)	12 (54.5)	0.43
Yes	26 (51.0)	10 (45.5)	
Coronary heart disease	21 (41.2)	9 (40.9)	0.98
Myocardial infarction	5 (9.8)	3 (13.6)	0.48
Stroke	6 (11.8)	5 (22.7)	0.23
COPD	8 (15.7)	2 (9.1)	0.45
Arterial aneurysm family history			
No	49 (96.1)	16 (72.7)	<0.05
Yes	2 (3.9)	6 (27.3)	
**Biochemical and hematological data, mean ± SD**			
White blood cells ($\times 10^9$/L)	6.9 ± 2.5	7.9 ± 2.4	0.11
Neutrophils ($\times 10^9$/L)	4.3 ± 1.5	4.3 ± 1.6	0.93
C-reactive protein (mg/L)	3.8 ± 2.0	4.5 ± 1.6	0.12
Creatinine (μmol/L)	83.0 ± 20.8	84.9 ± 23.6	0.74
Blood urea nitrogen (mmol/L)	5.9 ± 1.5	5.6 ± 1.2	0.49
Cholesterol (mg/dL)	148.2 ± 25.5	143.9 ± 29.9	0.53
Triglycerides (mg/dL)	147.9 ± 22.7	140.0 ± 29.3	0.22
ALT, U/L	19.7 ± 9.9	18.7 ± 8.0	0.67
AST, U/L	20.9 ± 9.6	22.9 ± 8.6	0.41
Glucose, mg/dL	95.2 ± 35.0	98.5 ± 22.5	0.69
D-dimer, ng/ml	115.1 ± 45.0	118.0 ± 54.5	0.81

COPD, chronic obstructive pulmonary disease; SD, standard deviation; ALT, alanine aminotransferase; AST, aspartate transaminase

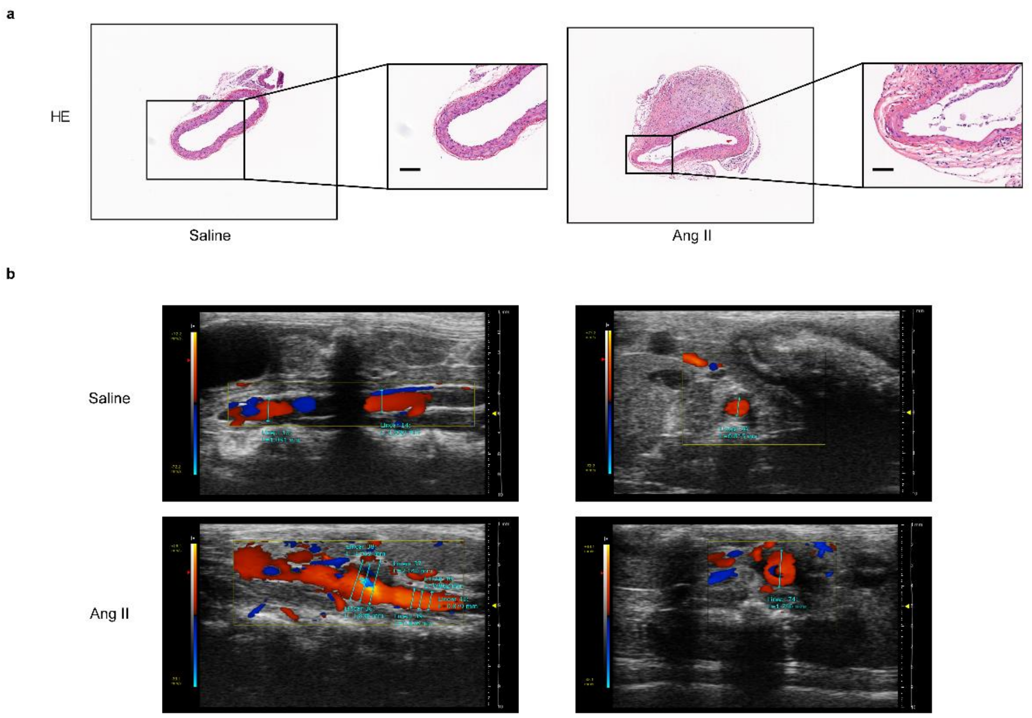

Supplementary Fig. 1 Pathological characteristics of AAA mice model

a Representative images of H&E staining for the tissue samples of AAA models and controls. Scale bar = 100 μm. **b** Representative images of Doppler ultrasound of AAA models and controls.

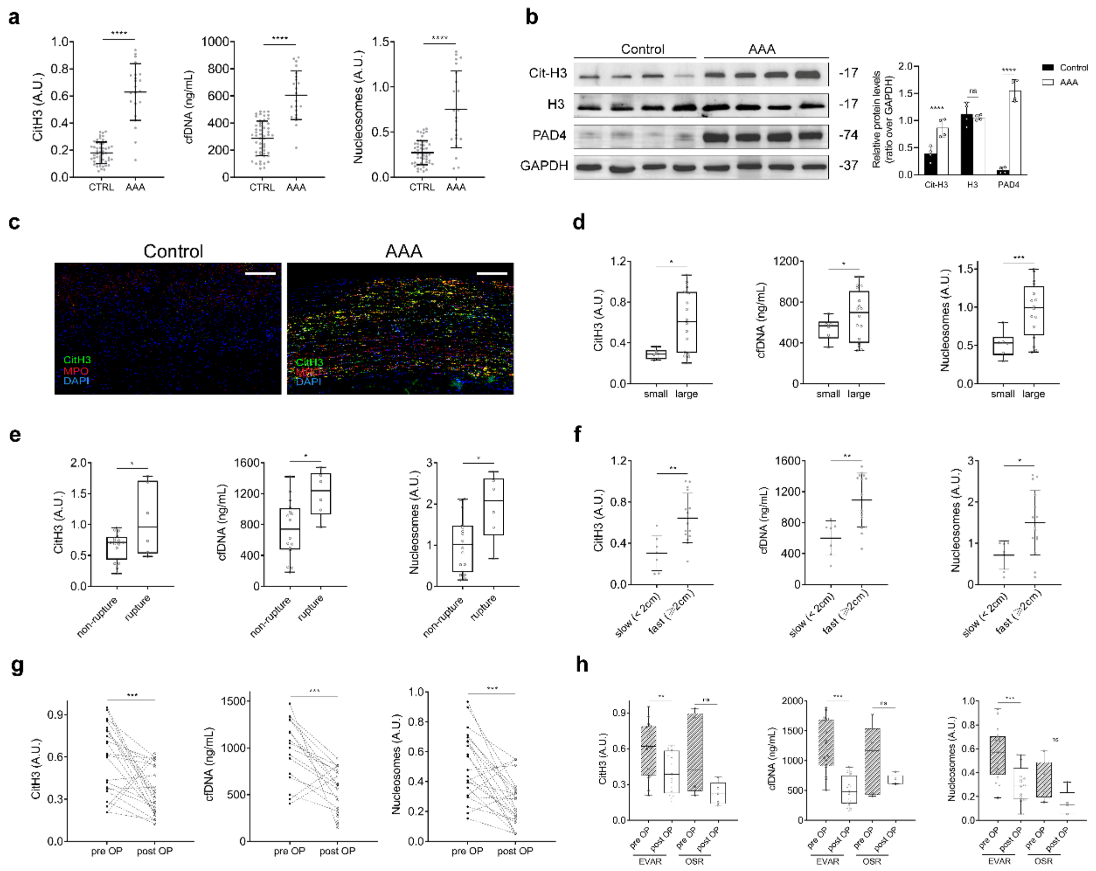

Supplementary Fig. 2 Increased NET release is associated with poor clinical outcomes in patients with AAA

a The levels of NETs markers, including Cit-H3, cfDNA, and nucleosomes in plasma of AAA patients (n = 22) vs controls (n = 51). Student's t test. **b** Western blot for Cit-H3, H3, and PAD4 in tissue samples of AAA (n = 4) vs controls (n = 4). Student's t test. **c** Representative images of immunofluorescence staining for MPO (red), Cit-H3 (green), and DAPI (blue) in tissue samples of AAA and controls. Scale bar = 250 µm. **d** Serum levels of NETs markers in patients with small (n = 6) vs large AAA (n = 16). Student's t test. **e** Serum levels of NETs markers in patients with unruptured (n = 16) vs ruptured AAA (n = 6). Student's t test. **f** Serum levels of NETs markers in AAA patients with fast (n = 15) vs slowly (n = 7) developing AAA. Fast = maximum

aneurysm diameter increased ≥ 2cm in a single year. Student's t test. **g, h** Pre- and postoperative serum levels of NETs markers in AAA patients received endovascular therapy (n = 17) or open surgical repair (n = 5) *P < 0.05 and **P < 0.01, Student's t test. For all subfigures: ns: P > 0.05, *P < 0.05, **P<0.01, ***P<0.001, ****P<0.0001, data are given as mean ± SD.

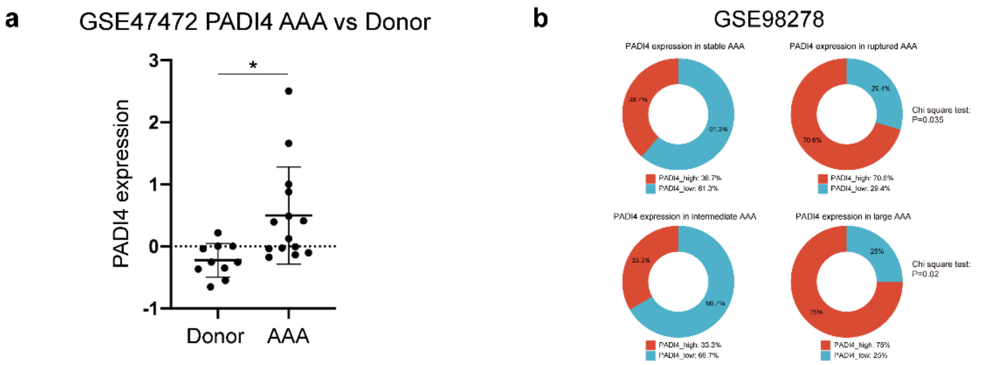

Supplementary Fig. 3 Expression of NETs-related indicators in the AAA-related GEO dataset

a PADI4 expression in AAA samples vs donor samples from GSE47472 dataset. $*P < 0.05$. Student's t test. This dataset contained the gene expression profile of biopsies obtained from the neck of human AAAs. **b** Correlation between risk of rupture (stable vs ruptured) and aneurysm size (intermediate vs large) and PADI4 expression from GSE98278 dataset.

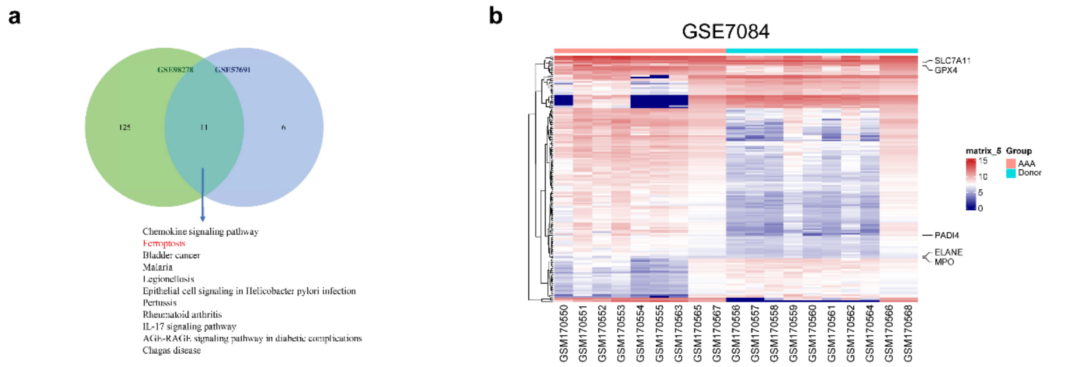

Supplementary Fig. 4 Expression of ferroptosis indicators in the AAA-related GEO dataset

a The Venn diagram obtained from the Kyoto Encyclopedia of Genes and Genomes (KEGG) enrichment analysis of two datasets (GSE98278 and GSE57691). **b** Heatmap of differential expressed genes screened from GSE7084 dataset showed the levels of NETs markers (PADI4, ELANE, and MPO) and ferroptosis markers (GPX4 and SLC7A11) in AAA samples vs donor samples.

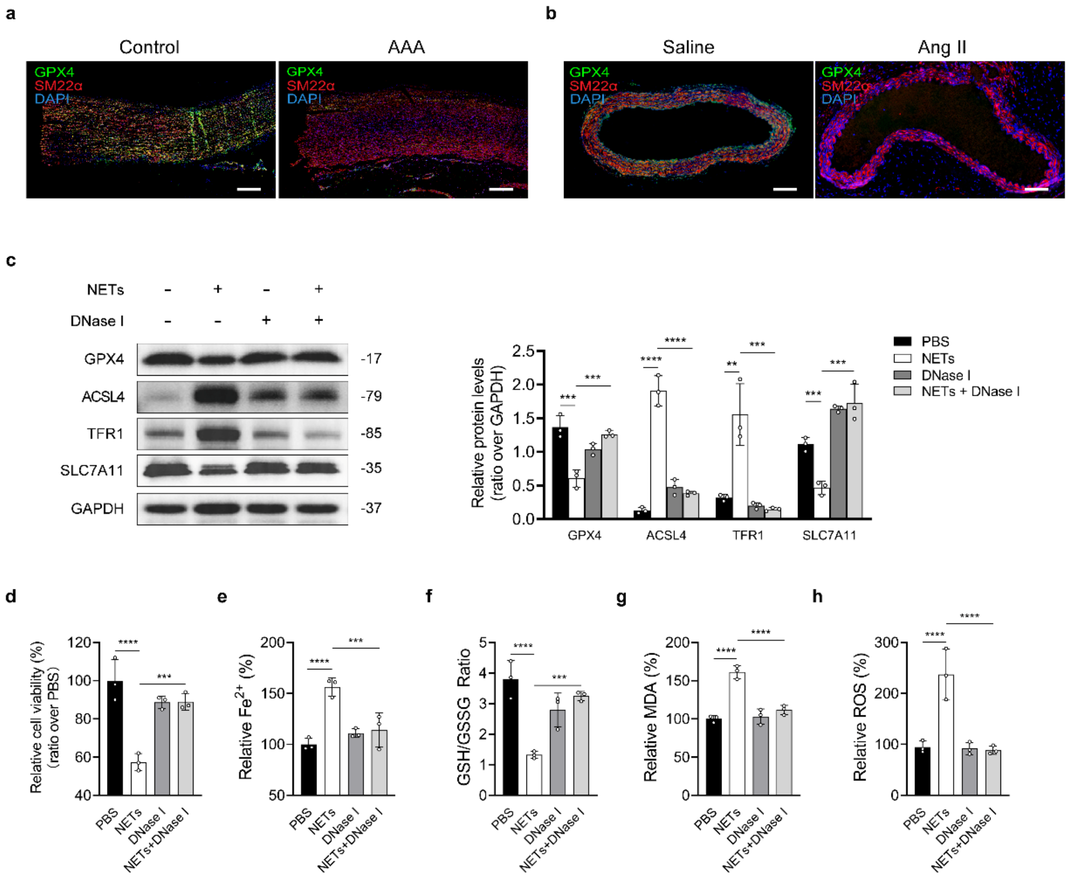

Supplementary Fig. 5 NETs promote ferroptosis of SMCs

a Representative images of immunofluorescence staining for SM22α (red), GPX4 (green), and DAPI (blue) in tissue samples of patients with AAA and controls. Scale bar = 250 μm. **b** Representative images of immunofluorescence staining for SM22α (red), GPX4 (green), and DAPI (blue) from the AAA mouse model and controls. Scale bar = 100 μm. **c** Western blot of GPX4, ACSL4, TFR1, and SLC7A11 in SMC treated with NETs for 8h, with or without DNase I (0.1 mg/mL) treatment. n = 3 in each group, one-way ANOVA followed by the SNK-q post hoc test. **d** Cell viability was assayed in SMC treated with NETs for 8h, with or without DNase I treatment. n = 3 in each group, one-way ANOVA followed by the SNK-q post hoc test. **e-h** The relative values of Fe^{2+}, GSH/GSSG ratio, MDA, and lipid ROS was assayed. n = 3 in

each group, one-way ANOVA followed by the SNK-q post hoc test. For all subfigures: $^{**}P<0.01$, $^{***}P<0.001$, $^{****}P<0.0001$, data are given as mean ± SD.

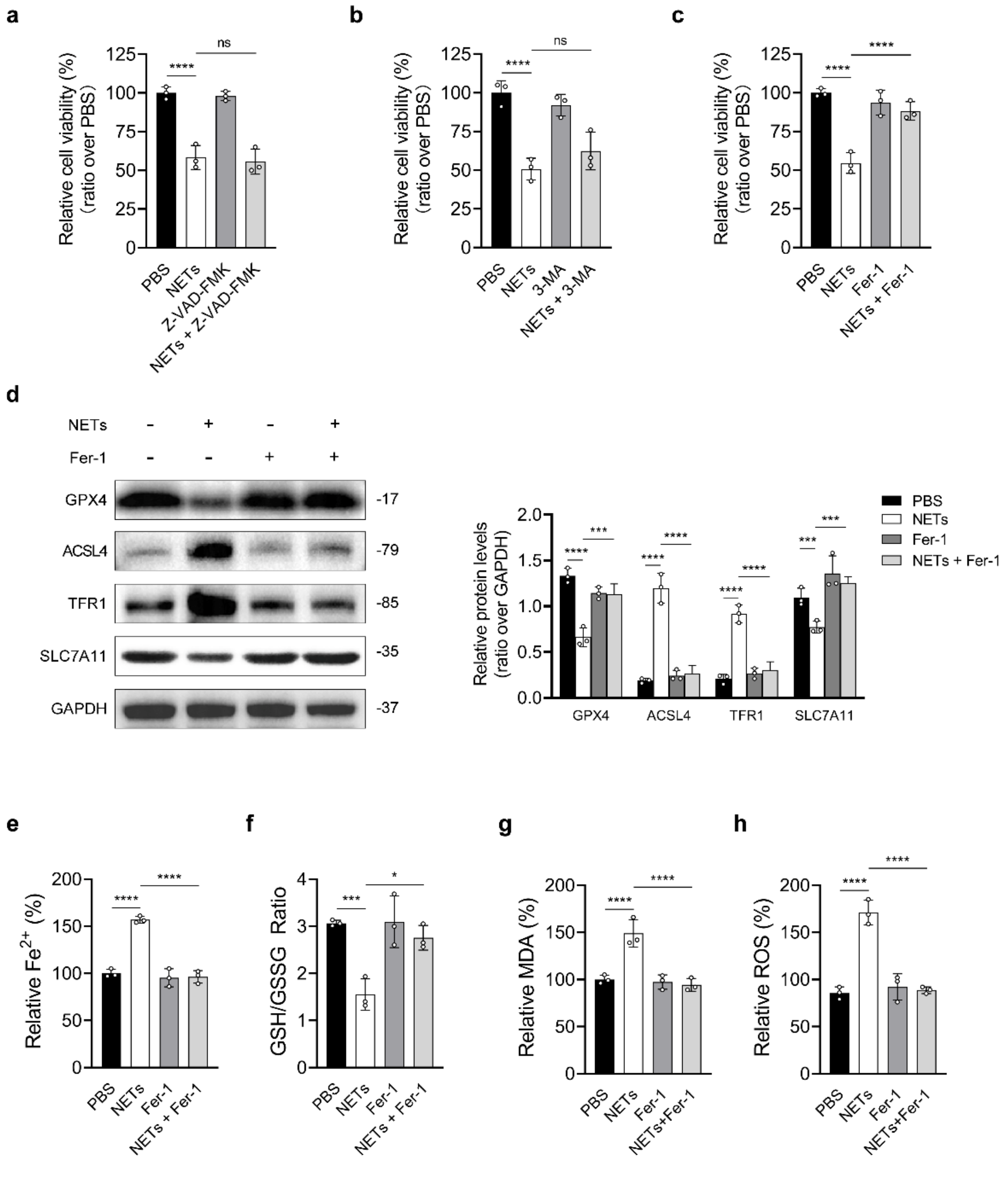

Supplementary Fig. 6 Fer-1 attenuates NETs-induced ferroptosis of SMCs

a Cell viability was assayed in SMCs treated with NETs for 8h, in the presence of Z-VAD-FMK (40 μM). n = 3 in each group, one-way ANOVA followed by the SNK-q post hoc test. **b** Cell viability was assayed in SMCs treated with NETs for 8h, in the presence of 3-MA (10 mM). n = 3 in each group, one-way ANOVA followed by the SNK-q post hoc test. **c** Cell viability was assayed in SMC treated with NETs for 8h, in the presence of Fer-1 (1 μM). n = 3 in each group, one-way ANOVA followed by the

SNK-q post hoc test. **d** Western blot of GPX4, ACSL4, TFR1, and SLC7A11 in SMC treated with NETs for 8h, with or without Fer-1 treatment. n = 3 in each group, one-way ANOVA followed by the SNK-q post hoc test. **e-h** The relative values of Fe^{2+}, GSH/GSSG ratio, MDA, and lipid ROS were assayed. n = 3 in each group, one-way ANOVA followed by the SNK-q post hoc test. For all subfigures: ns: $P > 0.05$, *$P < 0.05$, ***$P<0.001$, ****$P<0.0001$, data are given as mean ± SD.

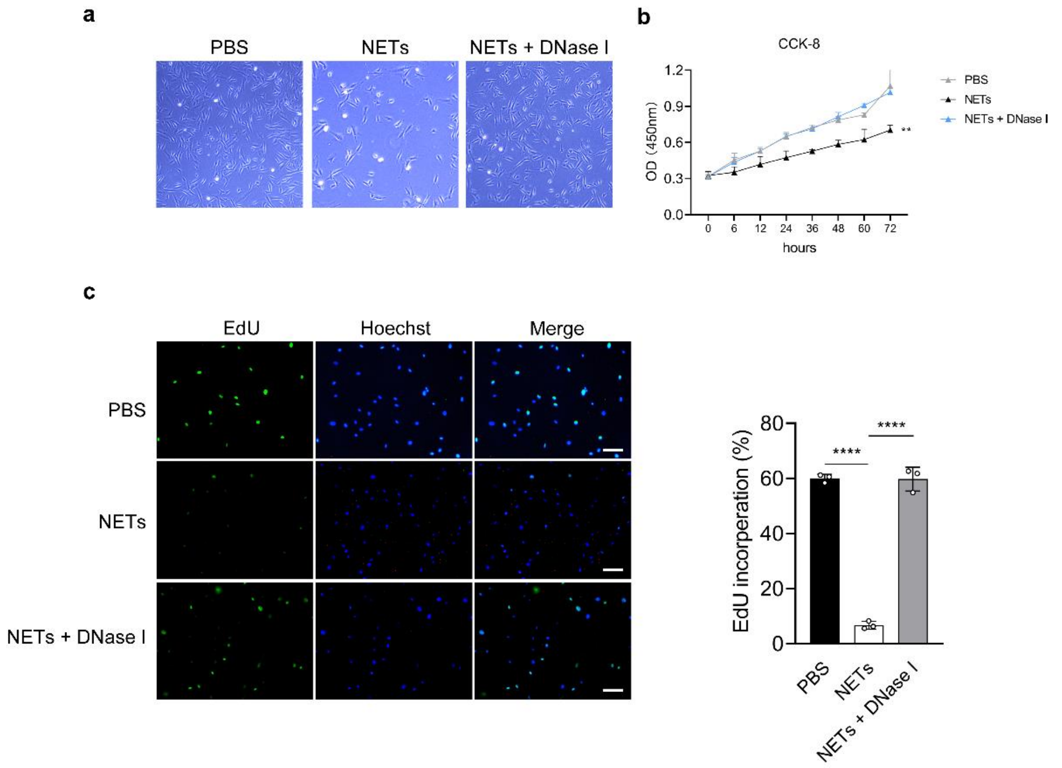

Supplementary Fig. 7 NETs reduce the proliferation of SMCs

a The confluence of SMCs in NETs and NETs + DNase I (0.1 mg/mL) groups under light microscope. **b** SMC proliferation was analyzed by CCK-8 assay. ** $P < 0.01$. n = 3 in each group. one-way ANOVA followed by the SNK-q post hoc test. **c** SMC proliferation was analyzed by EdU incorporation assay. n = 3 in each group. one-way ANOVA followed by the SNK-q post hoc test. Scale bar = 50 µm. For all subfigures: **P<0.01, ****P<0.0001, data are given as mean ± SD.

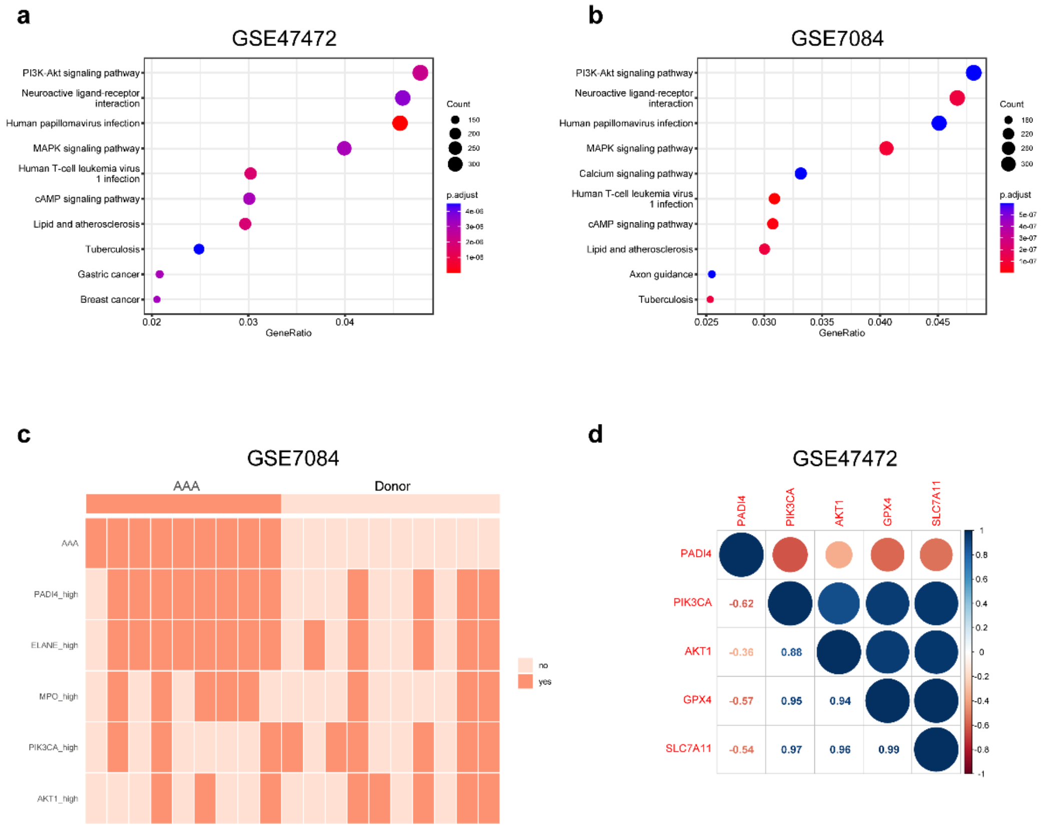

Supplementary Fig. 8 PI3K/AKT pathway is associated with NETs and ferroptosis in the AAA-related GEO dataset

a, b KEGG enrichment analysis of differential expressed genes screened in AAA samples comparing with donor from GSE47472 and GSE7084 dataset. **c** Levels of NETs markers (PADI4, ELANE and MPO), and PI3K/AKT pathway markers (PIK3CA and AKT1) from the GSE7084 dataset in relation to AAA prevalence. **d** Correlation between NETs marker (PADI4), PI3K/AKT pathway markers (PIK3CA and AKT1), and ferroptosis markers (GPX4 and SLC7A11) from GSE47472 dataset.

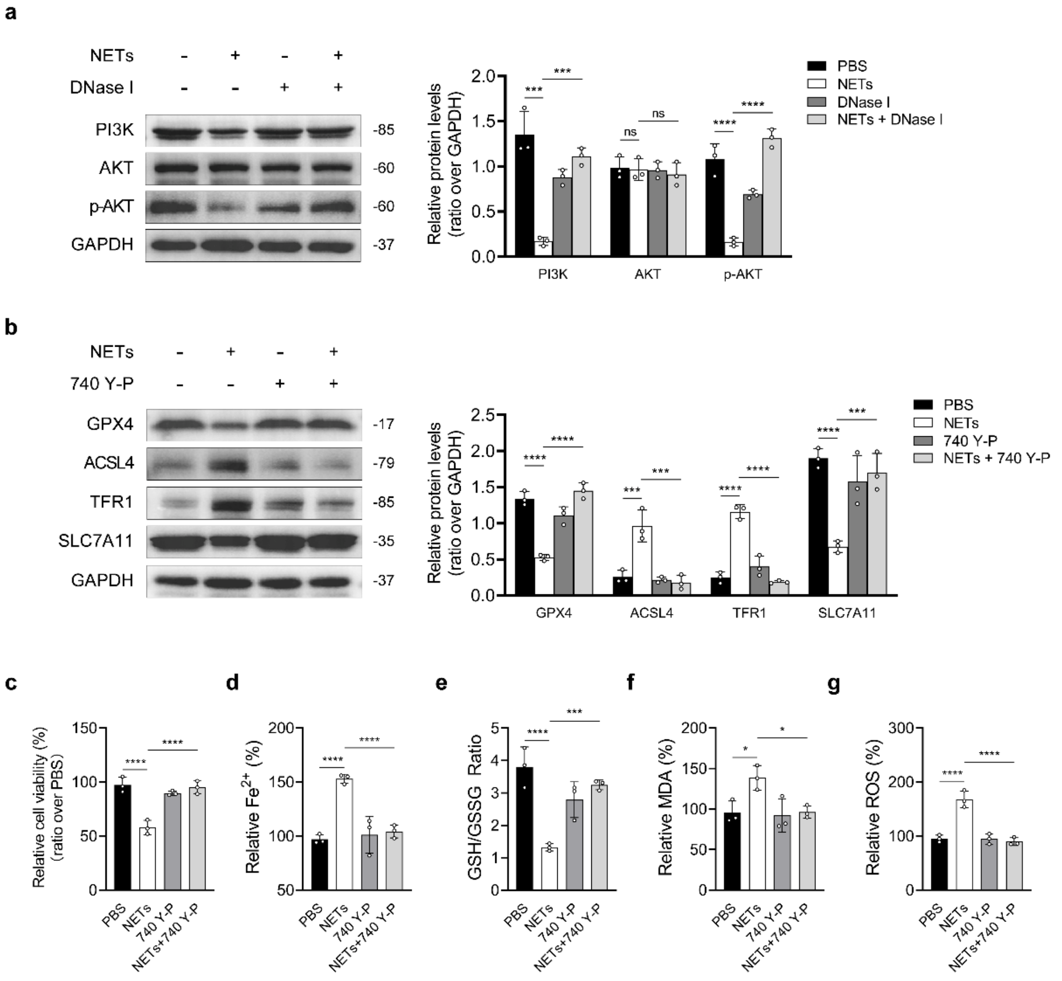

Supplementary Fig. 9 NETs promote SMC ferroptosis by inhibiting the PI3K/AKT pathway

a Western blot of PI3K, AKT, and p-AKT in SMCs treated with NETs for 8h, with or without DNase I (0.1 mg/mL) treatment. n = 3 in each group, one-way ANOVA followed by the SNK-q post hoc test. **b** Western blot of GPX4, ACSL4, TFR1, and SLC7A11 in SMCs treated with NETs for 8h, with or without 740 Y-P (50 μg/ml) treatment. n = 3 in each group, one-way ANOVA followed by the SNK-q post hoc test. **c** Cell viability was assayed in SMCs treated with NETs for 8h, with or without 740 Y-P treatment. n = 3 in each group, one-way ANOVA followed by the SNK-q post hoc test. **d-g** The relative values of Fe^{2+}, GSH/GSSG ratio, MDA, and lipid ROS were

assayed. n = 3 in each group, one-way ANOVA followed by the SNK-q post hoc test. For all subfigures: ns: $P > 0.05$, *$P < 0.05$, ***$P<0.001$, ****$P<0.0001$, data are given as mean ± SD.

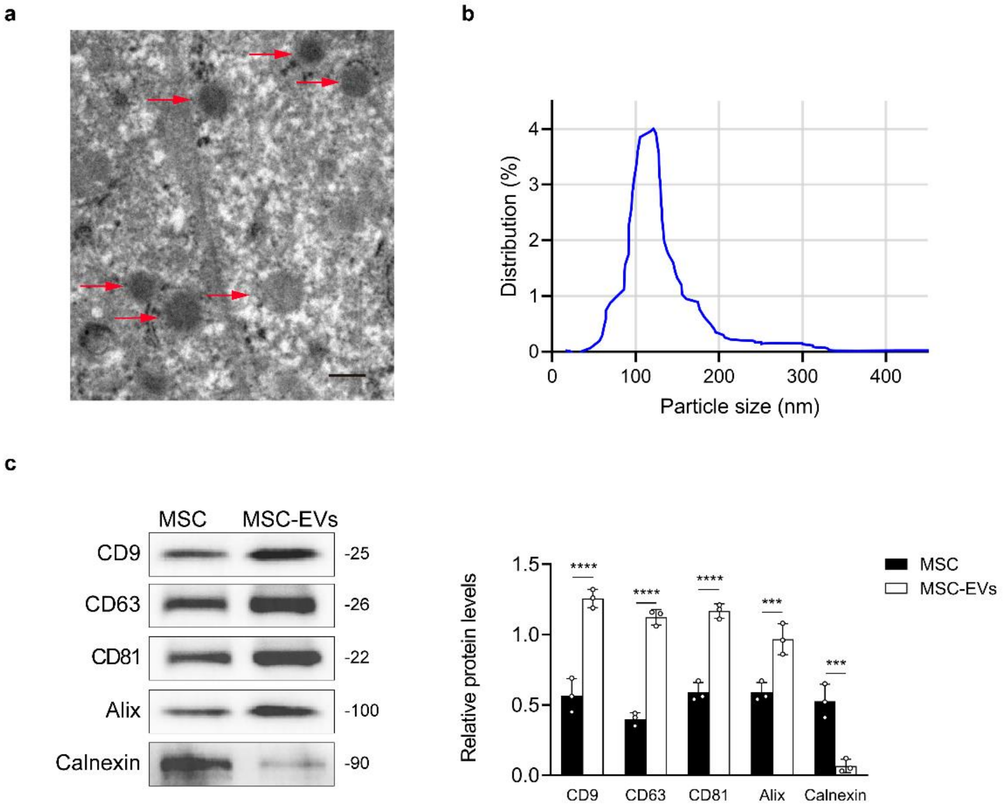

Supplementary Fig. 10 Characterization of MSC-EVs.

a Representative transmission electron microscopic images of MSC-EVs. Scale bar = 100 nm. **b** DLS analysis reveals the particle distribution of exosomes in various sizes. **c** Western blot for the biomarkers of EVs, including CD9, CD63, CD81, and Alix. Calnexin was used as a negative control. ***P<0.001, ****P<0.0001, data are given as mean ± SD.

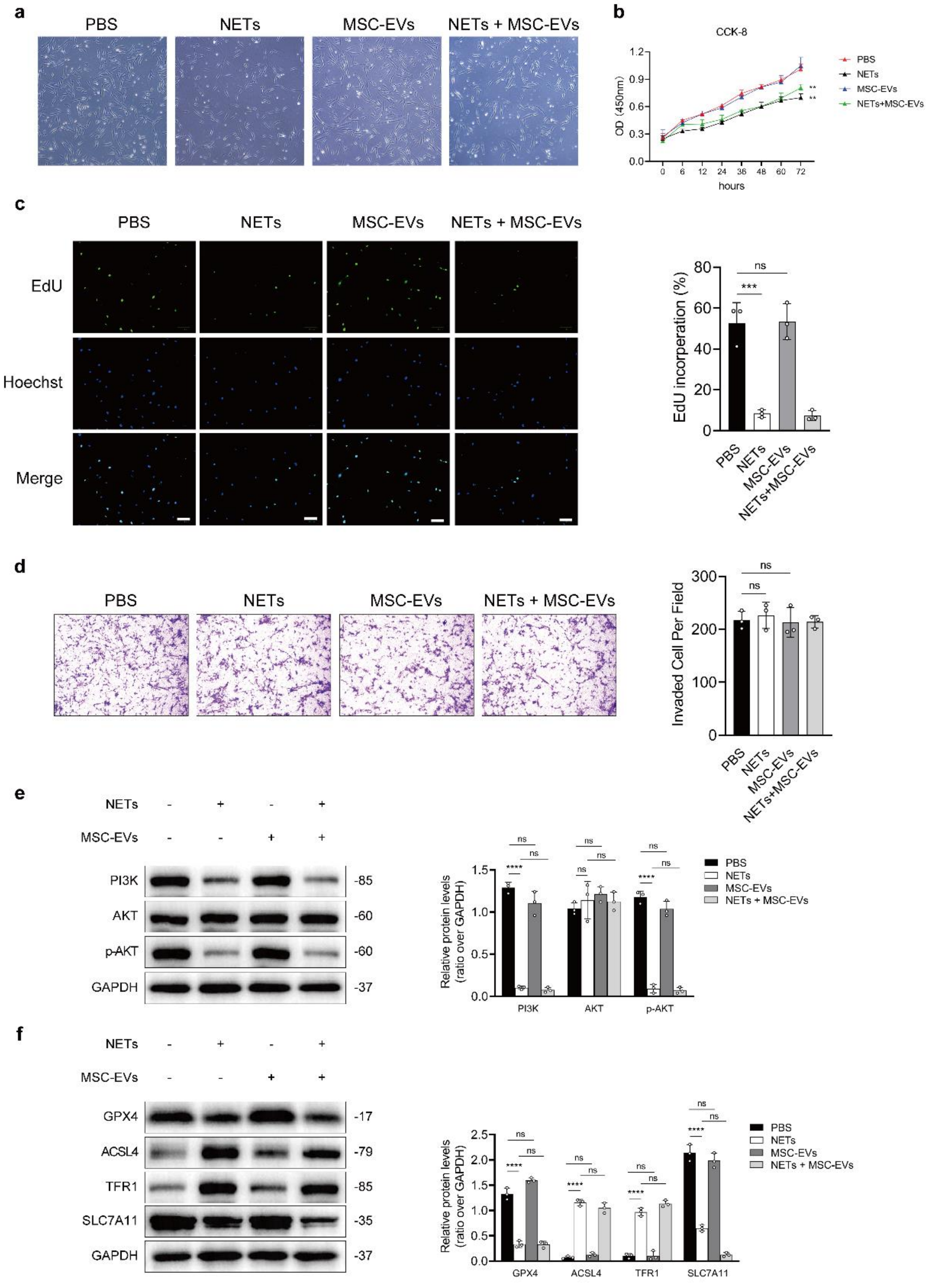

Supplementary Fig. 11 MSC-EVs have no effect on NETs-induced ferroptosis and inactivation of the PI3K/AKT pathway in SMCs

a The confluence of SMCs treated with NETs for 8h, with or without MSC-EVs (200 ng/μL) treatment under light microscope. **b** SMC proliferation was analyzed by CCK-8 assay. n = 3 in each group. one-way ANOVA followed by the SNK-q post hoc test. **c** SMC proliferation was analyzed by EdU incorporation assay. n = 3 in each group. **d** SMC migration was analyzed by transwell assay. n = 3 in each group. **e** Western blot of PI3K, AKT, and p-AKT in SMCs treated with NETs for 8h, with or without MSC-EVs (200 ng/μL) treatment. N = 3 in each group, one-way ANOVA followed by the SNK-q post hoc test. **f** Western blot of GPX4, ACSL4, TFR1, and SLC7A11 in SMCs treated with NETs for 8h, with or without MSC-EVs (200 ng/μL) treatment. N = 3 in each group, one-way ANOVA followed by the SNK-q post hoc test. For all subfigures: ns: $P > 0.05$, $^{**}P<0.01$, $^{***}P<0.001$, $^{****}P<0.0001$, data are given as mean ± SD.

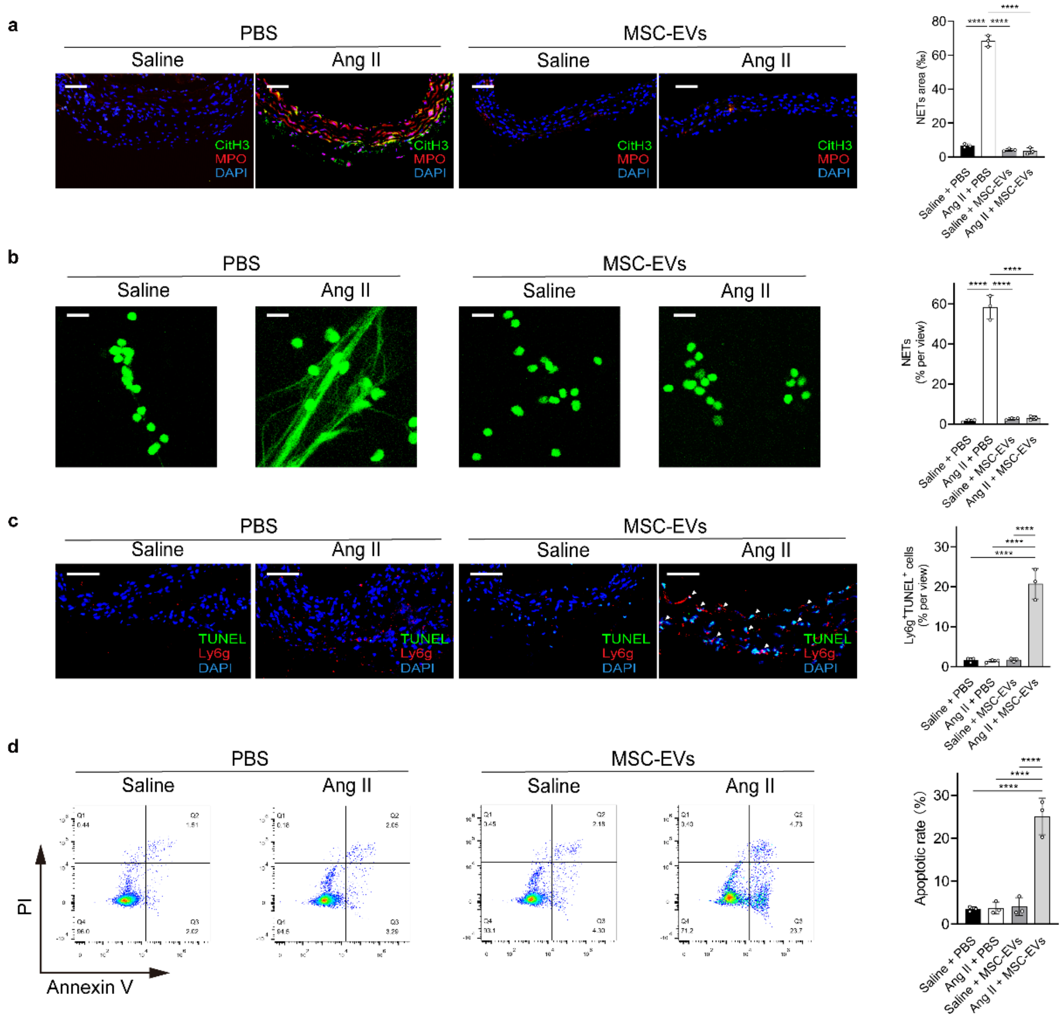

Supplementary Fig. 12 MSC-EVs reduce NET release and induce apoptosis in neutrophil in the Ang II-induced AAA model

a Representative images of immunofluorescence staining for MPO (red), Cit-H3 (green), and DAPI (blue) in aortic samples of mice. Scale bar = 150 µm. **b** The average of the number of NETs in the aortic tissues of mice. Scale bar = 10 µm. **c** Representative images of immunofluorescence staining for Ly6g (red), TUNEL (green), and DAPI (blue) in aortic samples of mice. Scale bar = 150 µm. Quantification of $Ly6g^{+}TUNEL^{+}$ cells (apoptotic neutrophils) in the aortic tissues of mice. **d** Annexin V/PI staining of $Ly6g^{+}$ cells from different groups by flow cytometry. n = 3 in each group, one-way ANOVA followed by the SNK-q post hoc

test. For all subfigures: n = 3 in each group, one-way ANOVA followed by the SNK-q post hoc test. ****P<0.0001, data are given as mean ± SD.

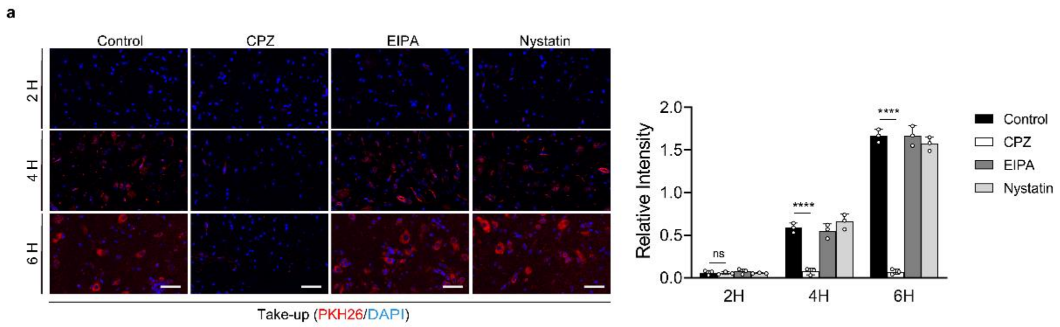

Supplementary Fig. 13 MSC-EVs are internalized by neutrophils via the clathrin-mediated endocytic pathway

a Neutrophils were incubated with signal inhibitors CPZ, EIPA, or nystatin for 2h before the incubation with MSC-EVs. Neutrophils incubated with 200 ng/μL PKH26-labeled MSC-EVs for the indicated times, and the uptake of MSC-EVs was detected by fluorescence microscopy. Scale bar = 50 nm. For all subfigures: ns: $P > 0.05$, ****$P<0.0001$, data are given as mean ± SD.

Uncut_gel_blot

Fig.1a

CitH3

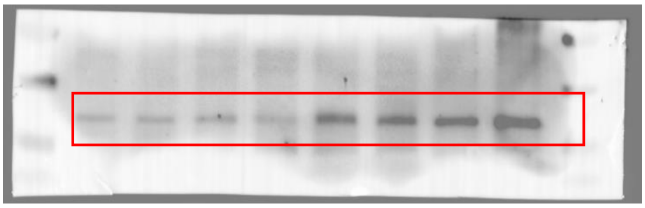

-17KDa

H3

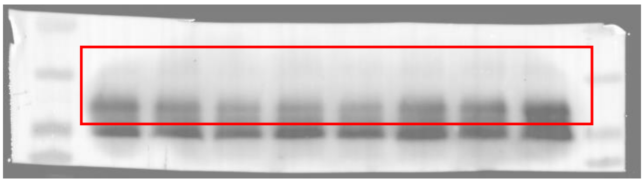

-17KDa

PAD4

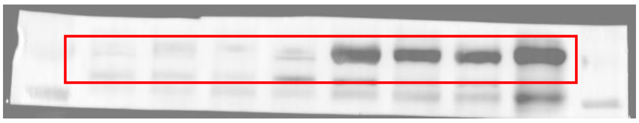

-74KDa

GAPDH

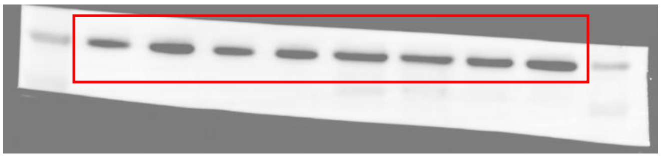

-37KDa

Fig.1c

CitH3

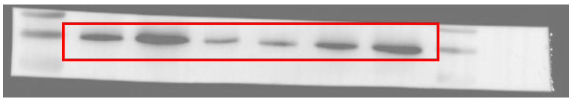

-17KDa

H3

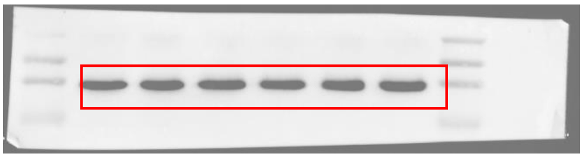

-17KDa

PAD4

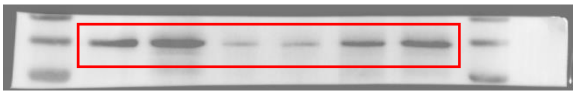

-74KDa

GAPDH

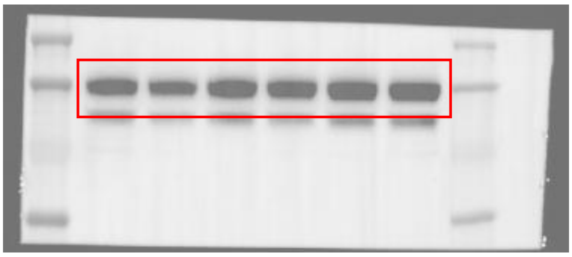

-37KDa

Fig.2a

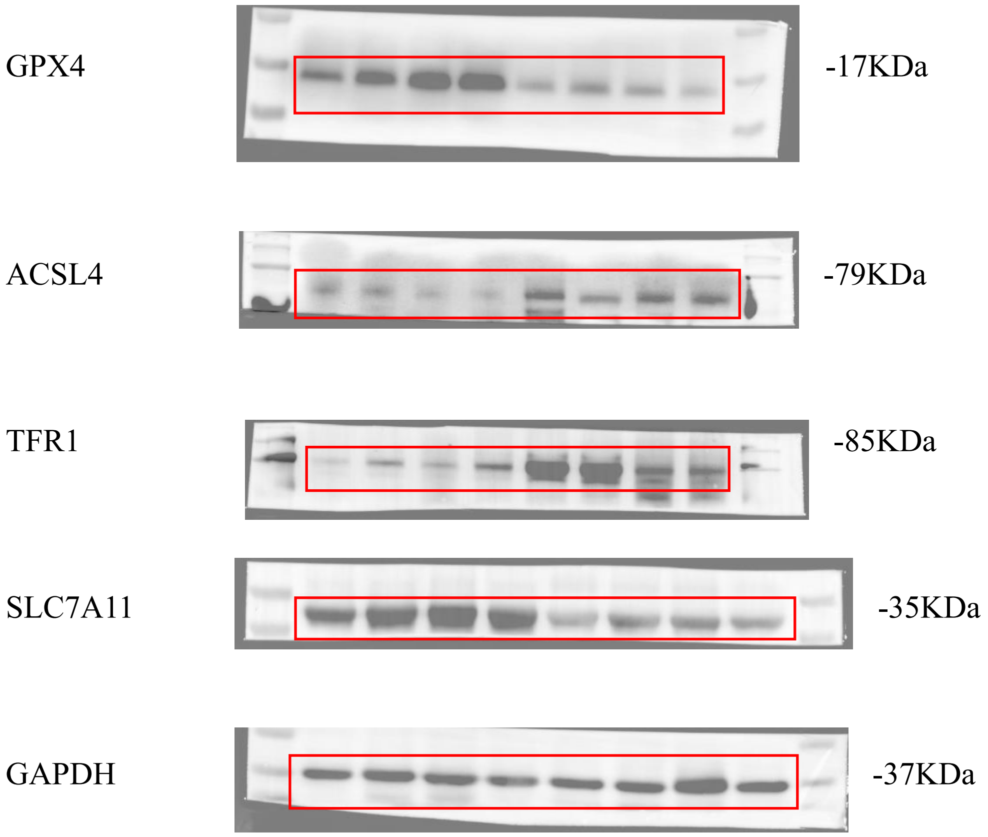
GPX4
-17KDa
ACSL4
-79KDa
TFR1
-85KDa
SLC7A11
-35KDa
GAPDH
-37KDa

Fig.2b

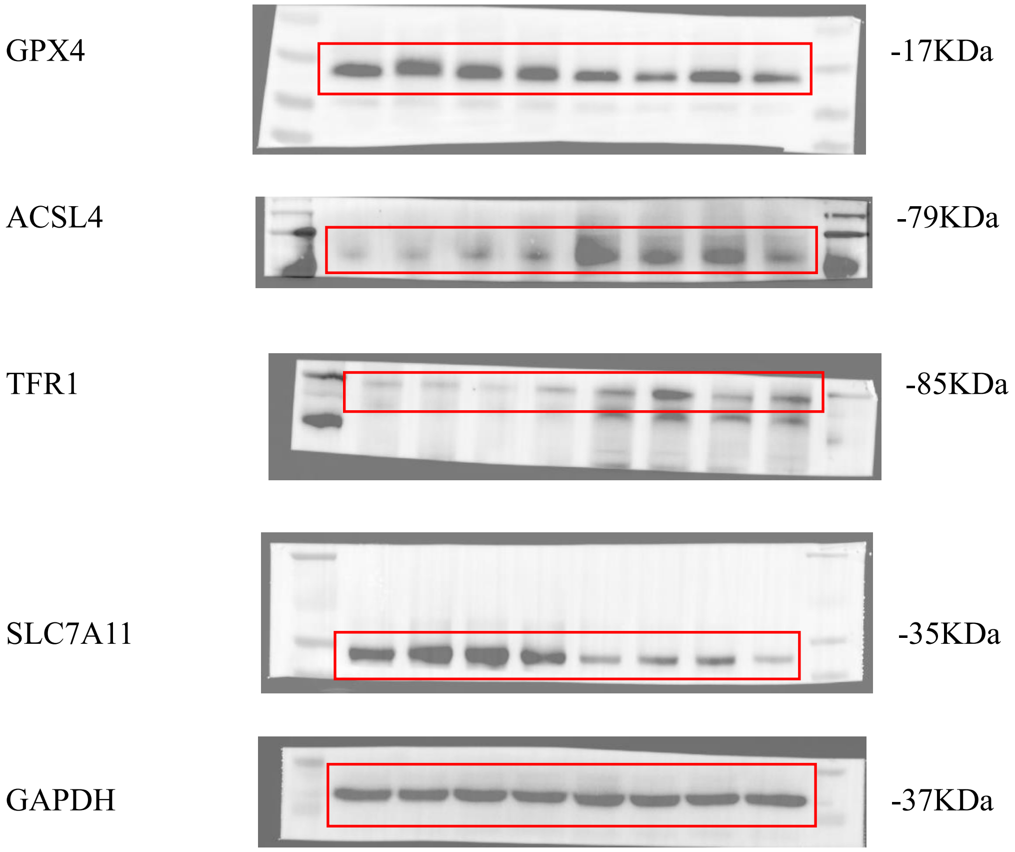
GPX4
-17KDa
ACSL4
-79KDa
TFR1
-85KDa
SLC7A11
-35KDa
GAPDH
-37KDa

Fig.2e

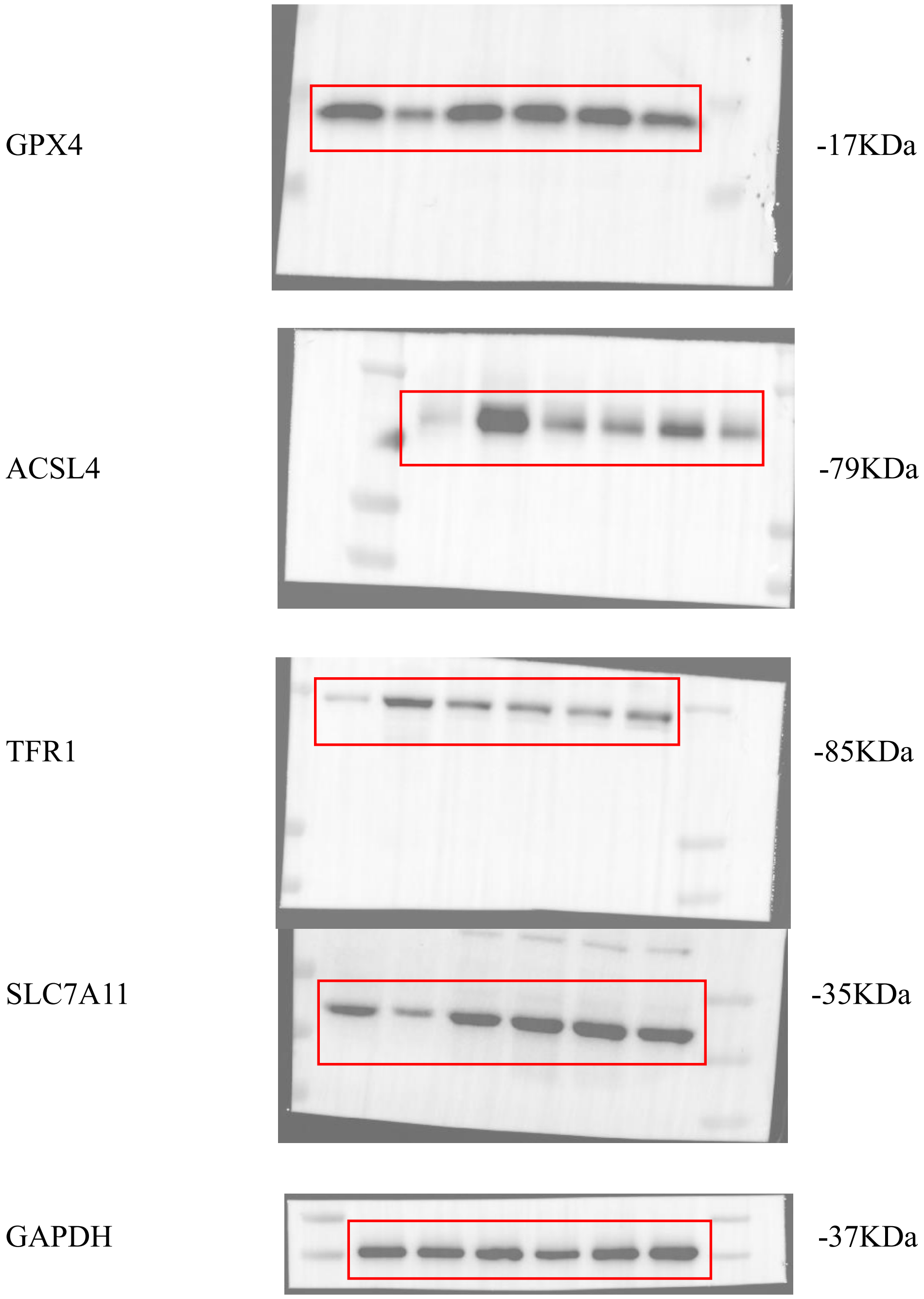
GPX4
-17KDa
ACSL4
-79KDa
TFR1
-85KDa
SLC7A11
-35KDa
GAPDH
-37KDa

Fig.2f

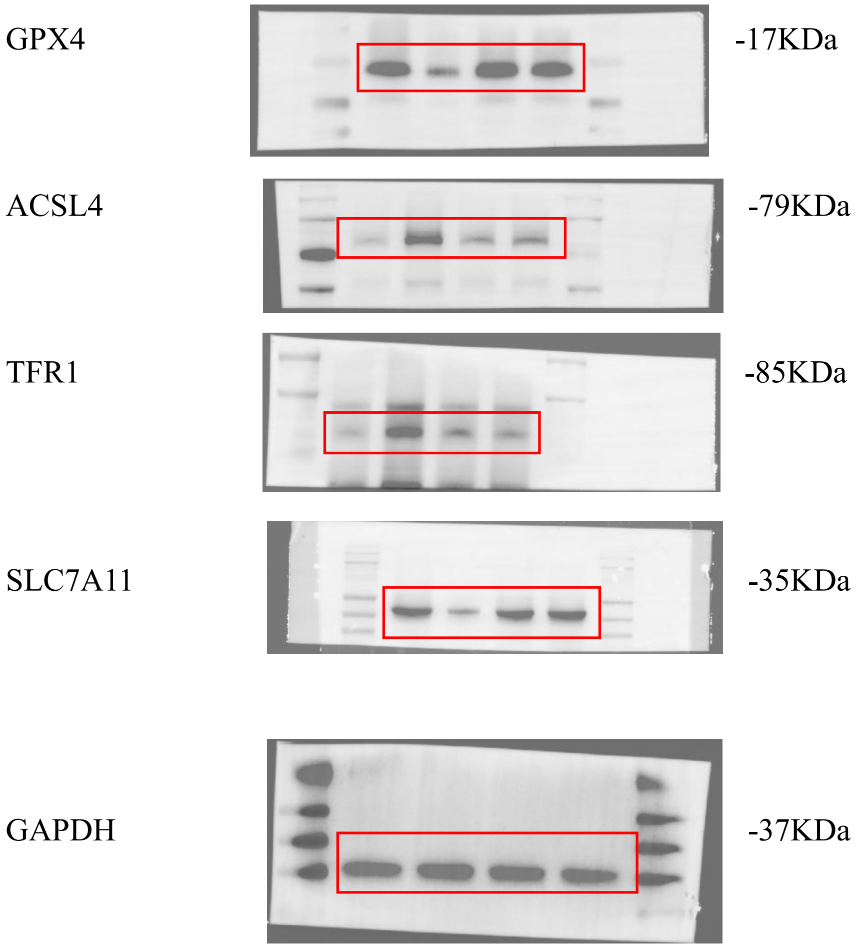
GPX4
-17KDa
ACSL4
-79KDa
TFR1
-85KDa
SLC7A11
-35KDa
GAPDH
-37KDa

Fig.3a

PI3K

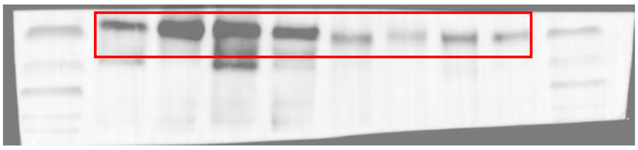

-85KDa

AKT

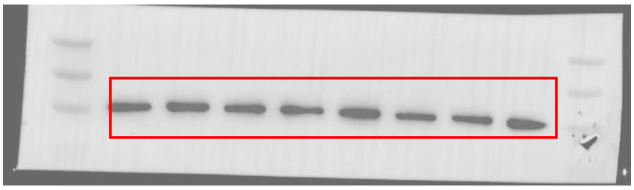

-60KDa

p-AKT

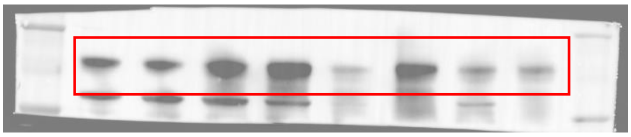

-60KDa

GAPDH

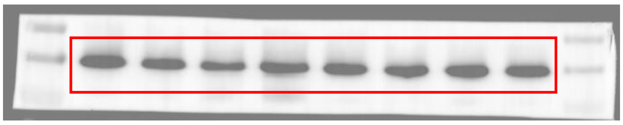

-37KDa

Fig.3c

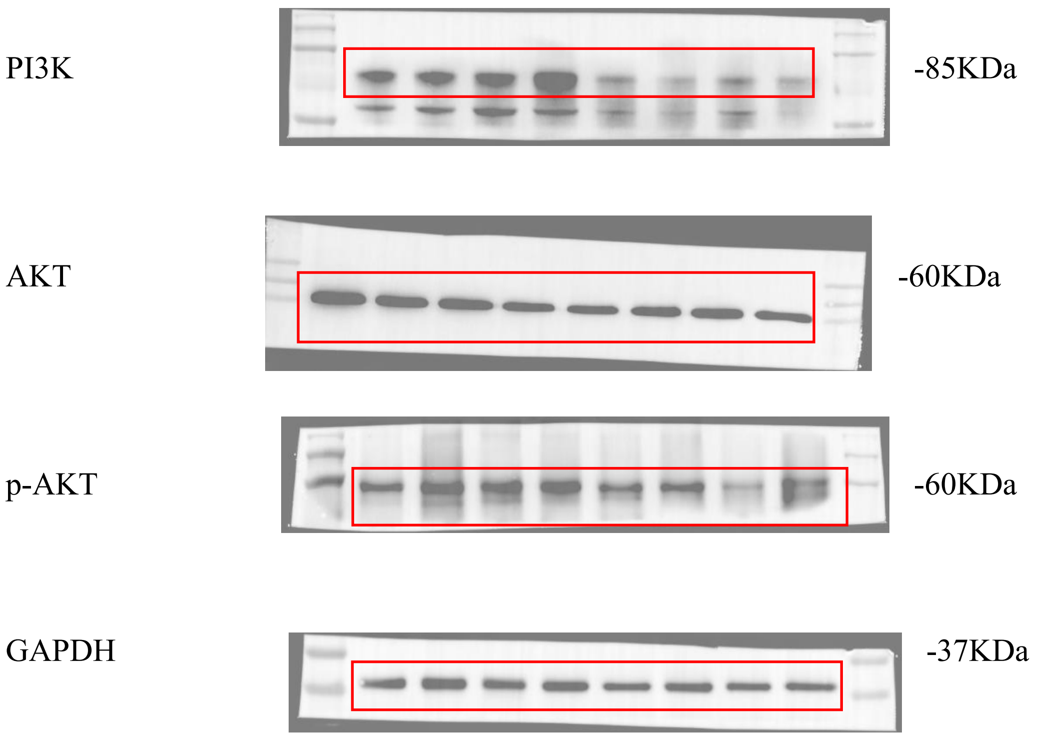

Fig.3e

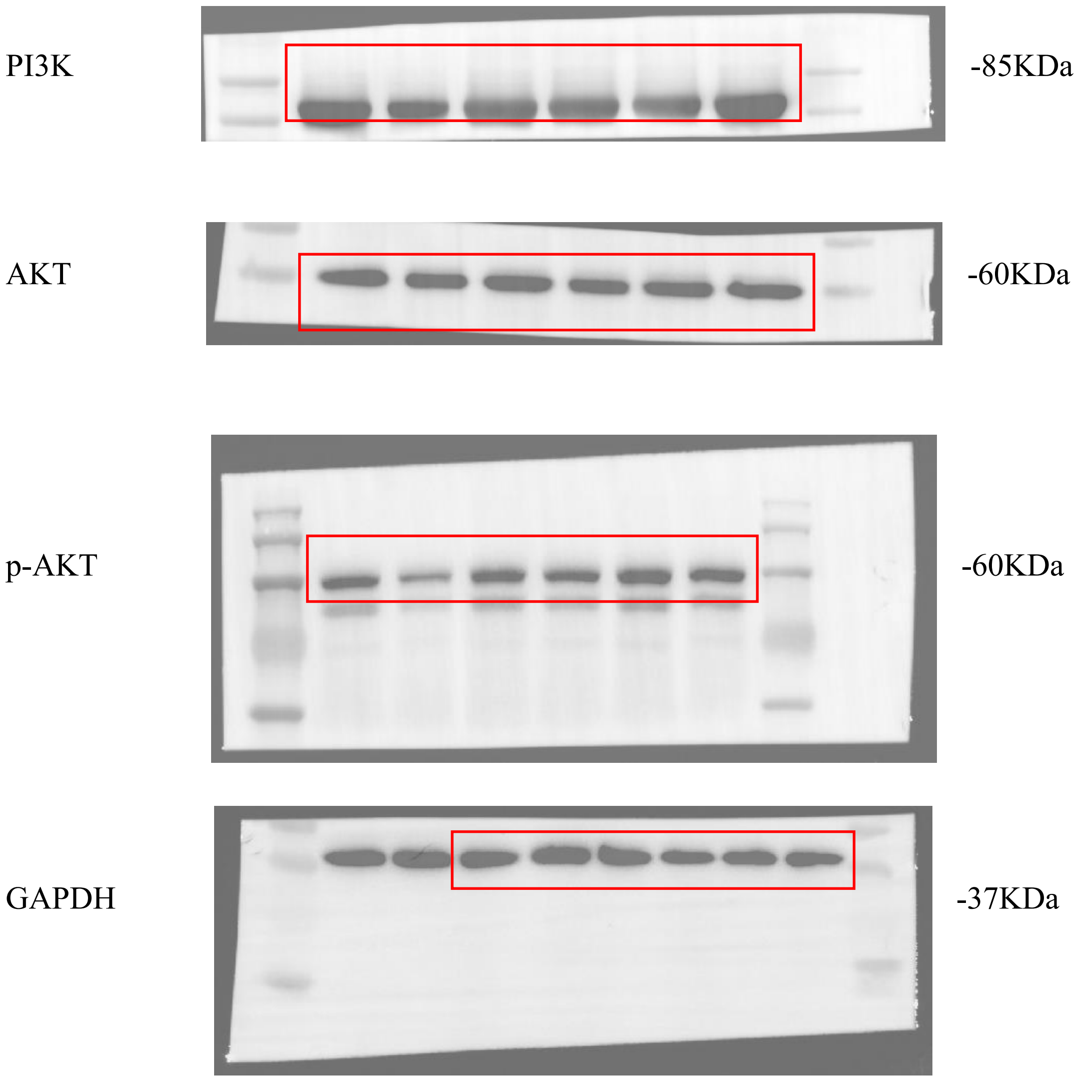

Fig.3f

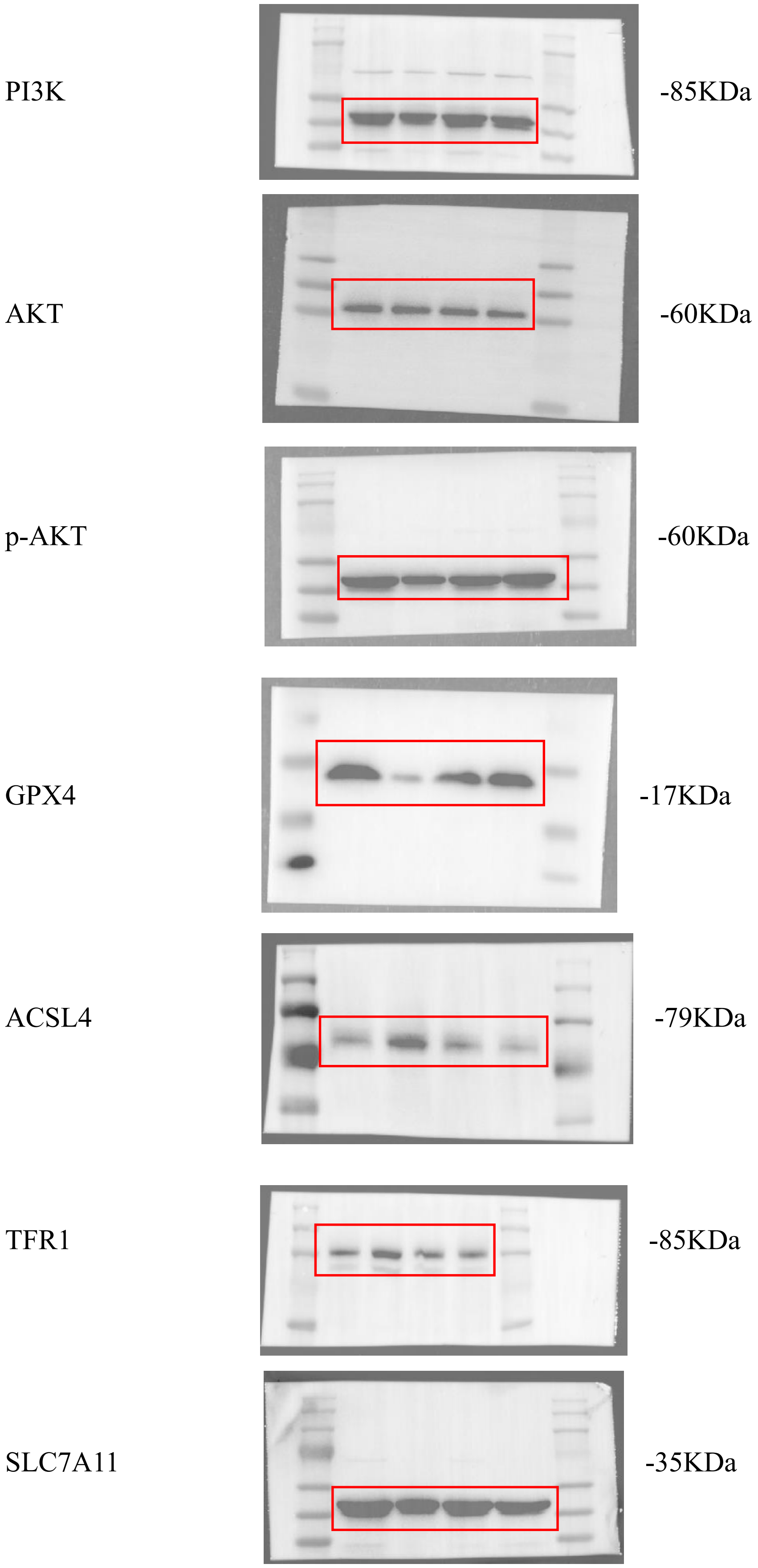
PI3K
-85KDa
AKT
-60KDa
p-AKT
-60KDa
GPX4
-17KDa
ACSL4
-79KDa
TFR1
-85KDa
SLC7A11
-35KDa

GAPDH

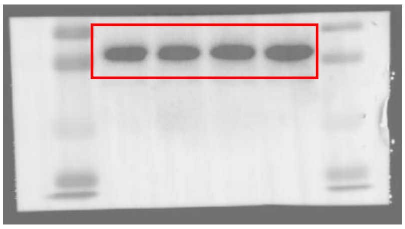

-37KDa

Fig.5a

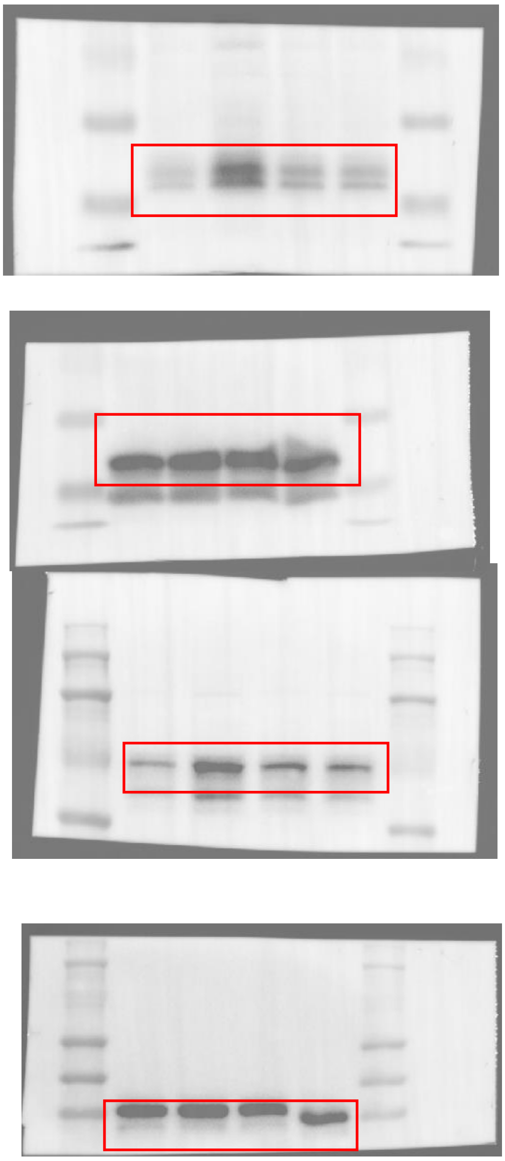

CitH3 -17KDa

H3 -17KDa

PAD4 -74KDa

GAPDH -37KDa

Fig.5d

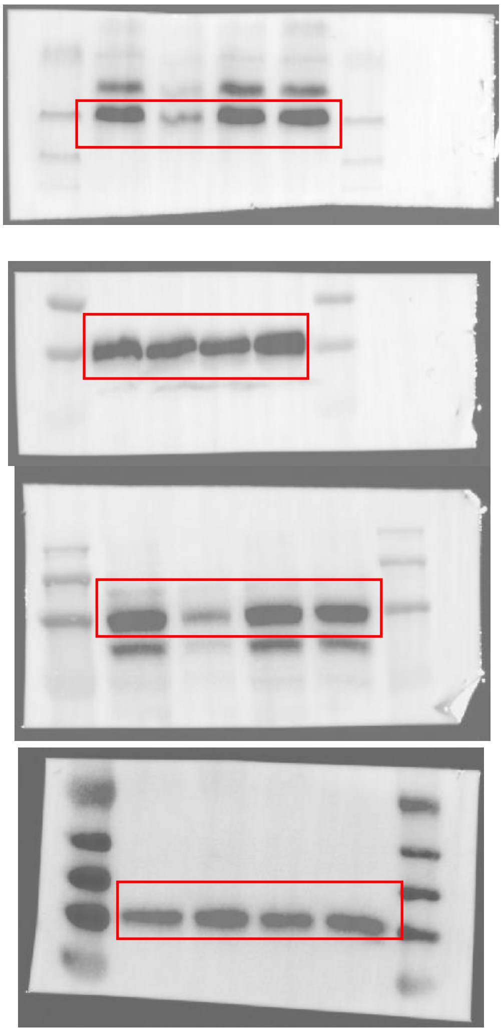

PI3K -85KDa

AKT -60KDa

p-AKT -60KDa

GAPDH -37KDa

Fig.5f

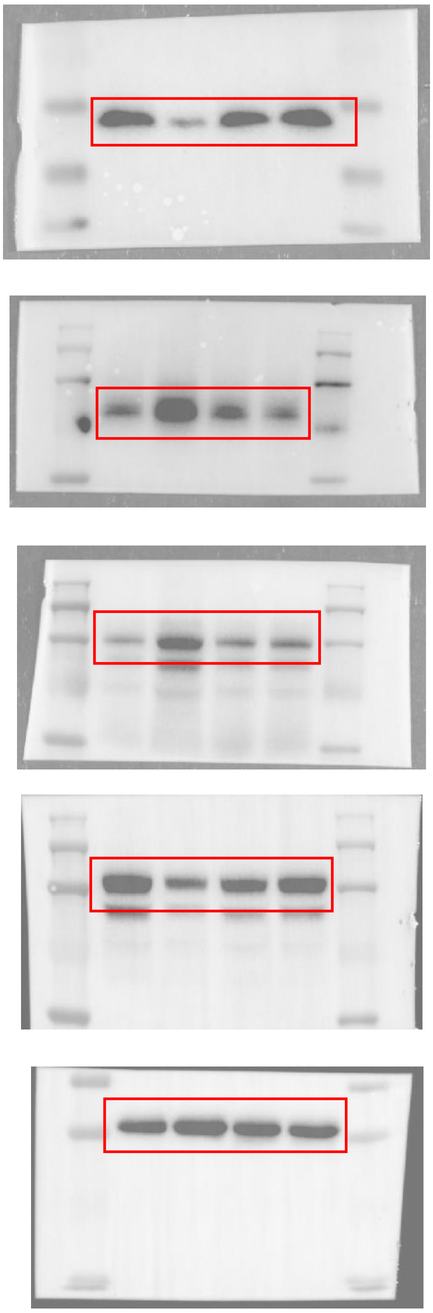

GPX4 -17KDa

ACSL4 -79KDa

TFR1 -85KDa

SLC7A11 -35KDa

GAPDH -37KDa

Fig.6a

PI3K

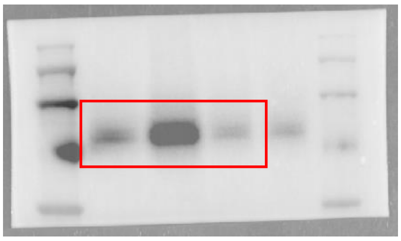

-85KDa

AKT

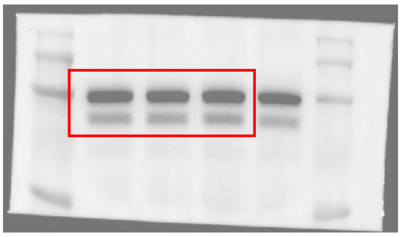

-60KDa

p-AKT

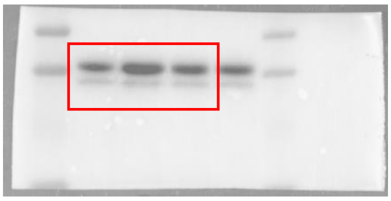

-60KDa

GAPDH

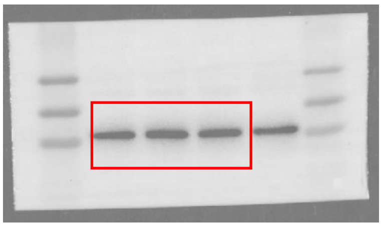

-37KDa

Supplementary Figure 2b

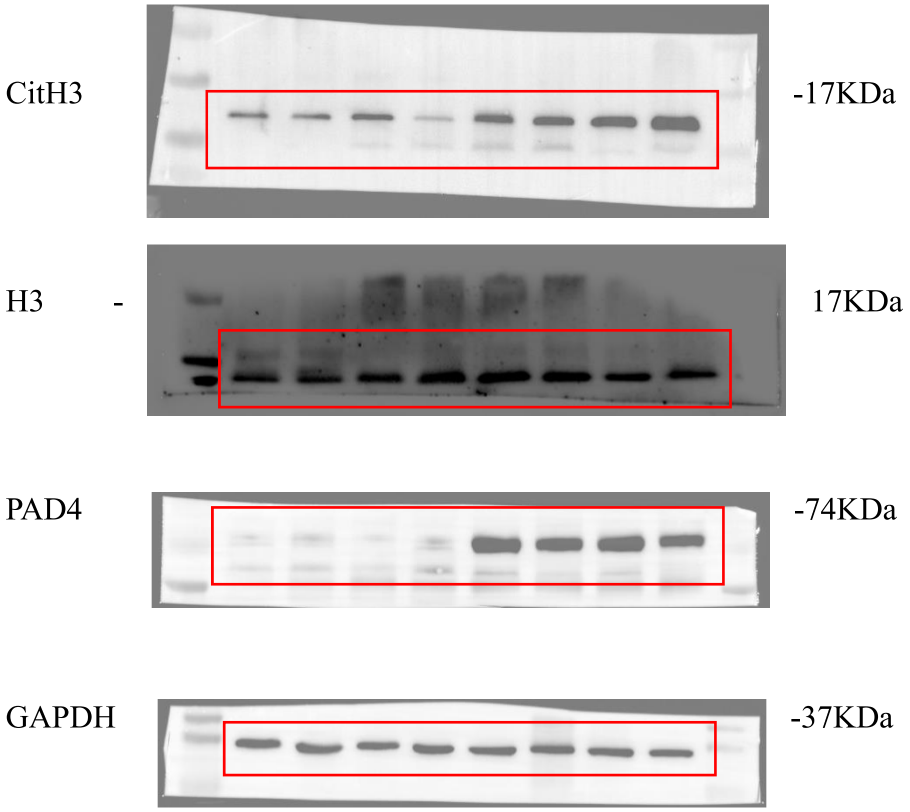

Supplementary Figure 5c

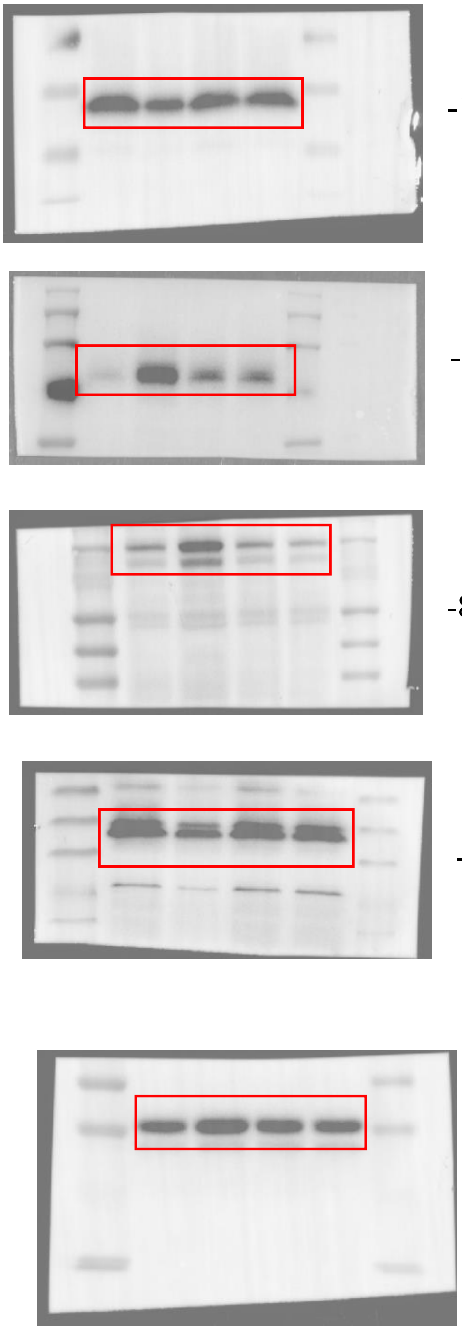

GPX4 -17KDa

ACSL4 -79KDa

TFR1 -85KDa

SLC7A11 -35KDa

GAPDH -37KDa

Supplementary Figure 6d

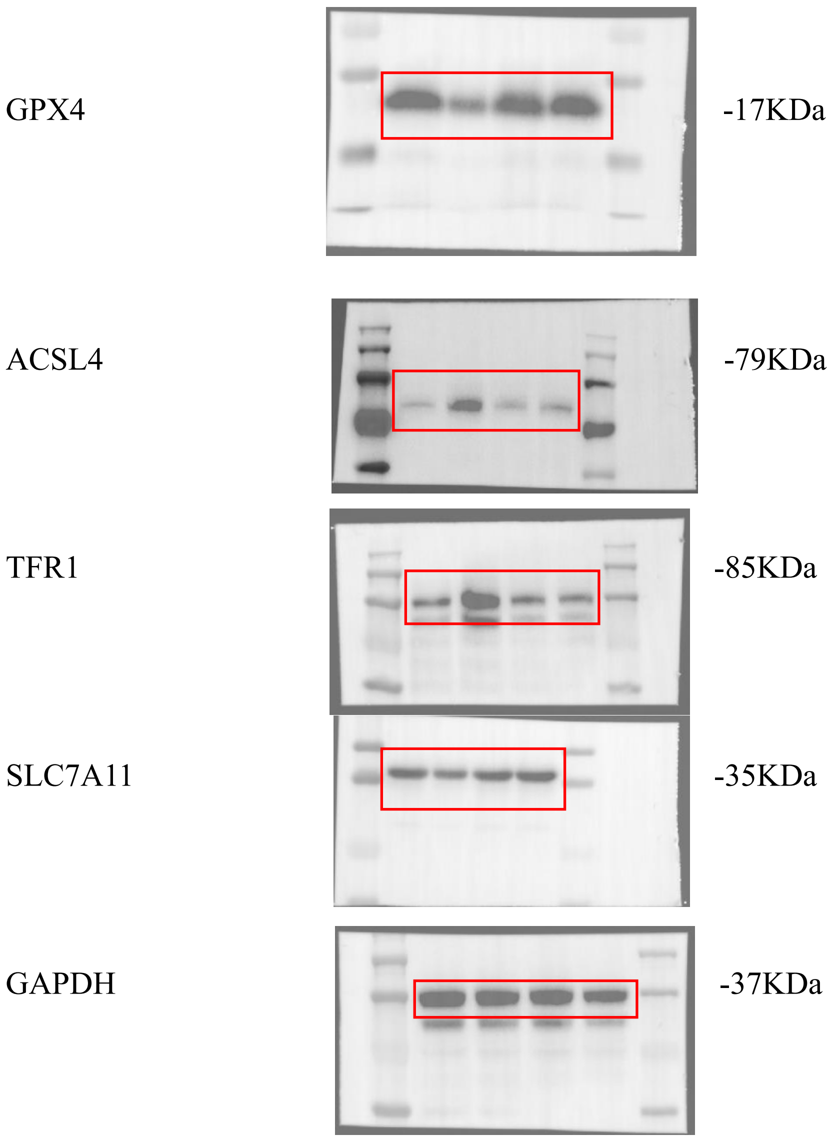

Supplementary Figure 9a

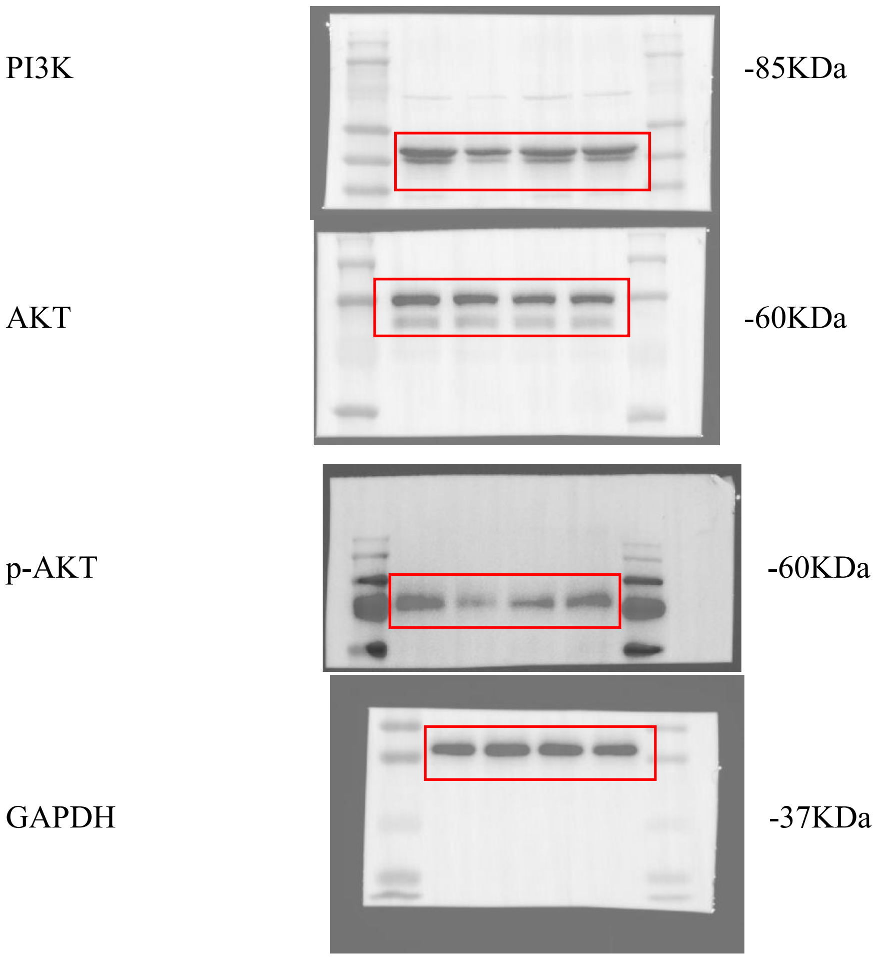

Supplementary Figure 9b

GPX4

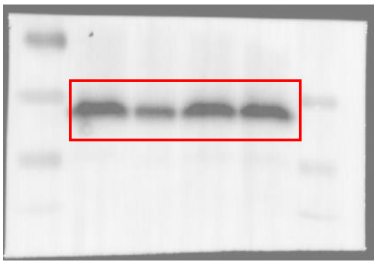

-17KDa

ACSL4

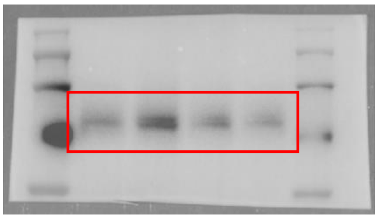

-79KDa

TFR1

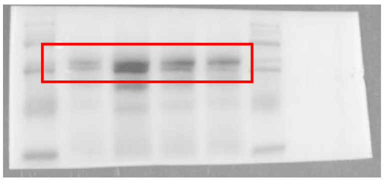

-85KDa

SLC7A11

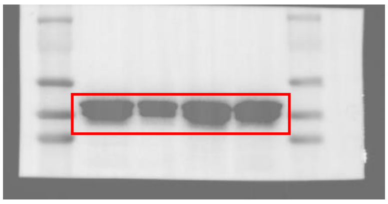

-35KDa

GAPDH

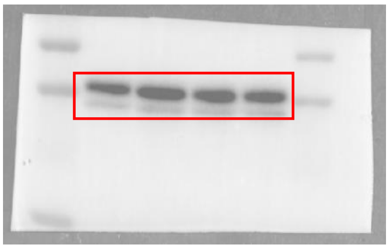

-37KDa

Supplementary Figure 10c

CD9

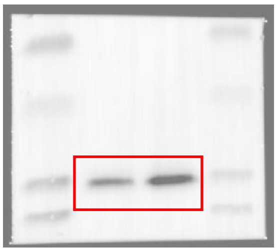

-25KDa

CD63

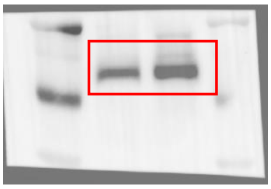

-26KDa

CD81

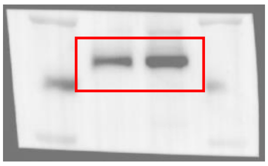

-22KDa

Alix

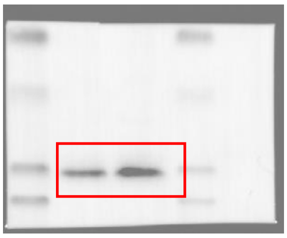

-100KDa

Calnexin

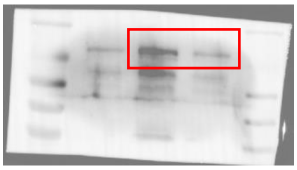

-90KDa

Supplementary Figure 11e

PI3K

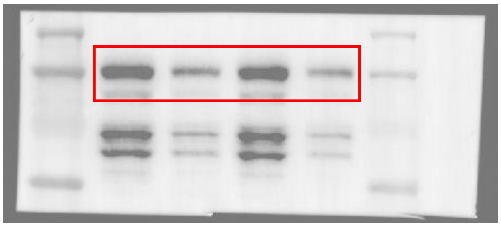

-85KDa

AKT

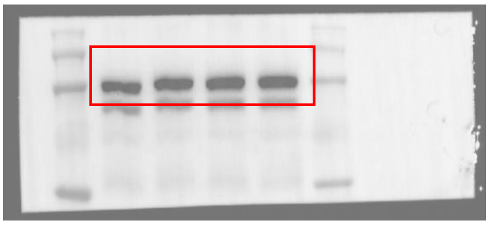

-60KDa

p-AKT

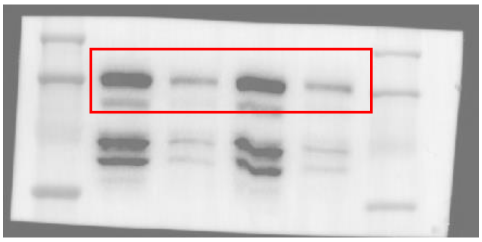

-60KDa

GAPDH

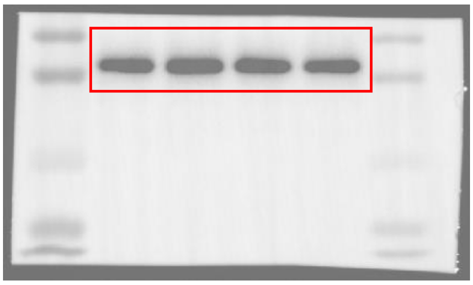

-37KDa

Supplementary Figure 11f

GPX4

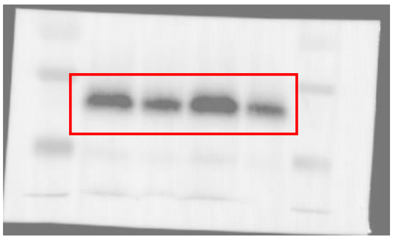

-17KDa

ACSL4

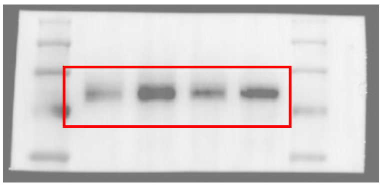

-79KDa

TFR1

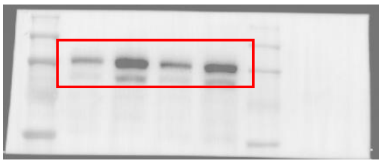

-85KDa

SLC7A11

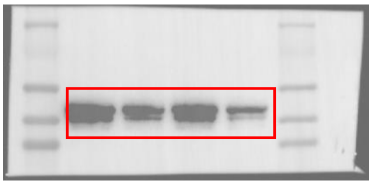

-35KDa

GAPDH

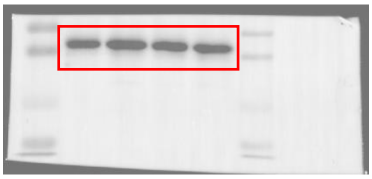

-37KDa
